# *Metarhizium anisopliae* Mitigates the Phytotoxicity of Lead and Nanoplastics on Rice by Modifying Physiological, Transcriptomic, Metabolomic Activities, and Soil Microbiome

**DOI:** 10.1002/advs.202521570

**Published:** 2026-02-06

**Authors:** Jing Peng, Qi Yan, Muhammad Umair Hassan, Muhammad Imran, Fasih Ullah Haider, Jianfeng Liang, Xingmin Wang, Shaukat Ali

**Affiliations:** ^1^ Department of Entomology College of Plant Protection South China Agricultural University Guangzhou China; ^2^ Engineering Research Center of Biological Control Ministry of Education, South China Agricultural University Guangzhou China; ^3^ Key Laboratory of Jiangxi Province for Biological Invasion and Biosecurity School of Life Sciences Jinggangshan University Ji'an China; ^4^ Jiangmen Laboratory of Carbon Science and Technology Hong Kong University of Science and Technology Jiangmen China; ^5^ Key Laboratory of Black Soil Conservation and Utilization Northeast Institute of Geography and Agroe‐cology Chinese Academy of Sciences Changchun China

**Keywords:** ABC transporters, lead toxicity, nanoplastics pollution, rice growth, soil microbiome, transcriptome

## Abstract

Polyethylene nanoplastics (NP) and lead (Pb) increasingly co‐occur in agriculture, where their effects exacerbate phytotoxic impacts. We tested whether the endophytic entomopathogenic fungus, *Metarhizium anisopliae*, can mitigate individual or combined stress of NP and Pb in rice by examining fungus‐soil‐plant mechanisms using physiological assays, transcriptomics, metabolomics, and rhizosphere microbiome profiling. Rice seedlings were grown under eight treatments (individual or combined stress of Pb and NP, with or without *M. anisopliae*). Individual and combined Pb and NP stress reduced seedling growth, chlorophyll content, and hormonal levels, while increasing oxidative damage. Pb and NP interactions showed synergistic toxicity, causing severe growth suppression and lipid peroxidation, and repressing photosynthesis and hormone‐related pathways. *M anisopliae* inoculation alleviated these effects and enhanced rice growth by reducing Pb uptake and translocation, restoring antioxidant and hormonal balance, and up‐regulating pathways including flavonoid biosynthesis, ABC transporters, and hormone signaling. Pb measurements showed fungal inoculation restricted Pb uptake as a protective mechanism. *M. anisopliae* reshaped the soil bacterial community, enriching taxa associated with plant growth promotion and contaminant tolerance. These findings identify *M. anisopliae* seed inoculation as a strategy to mitigate Pb and NP phytotoxicity in rice by integrating contaminant uptake control with plant and rhizosphere reprogramming.

## Graphical abstract

*Metarhizium anisopliae* alleviates the phytotoxic effects of polyethylene nanoplastics (NP) and lead (Pb) in rice by decreasing Pb uptake, restoring antioxidant and hormonal equilibrium, and promoting growth. Additionally, the fungus modifies the rhizosphere microbiota, enhancing both contaminant tolerance and plant growth, thereby effectively mitigating the stress induced by individual and combined NP and Pb exposure.

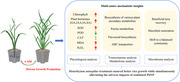

## 1 Introduction

Plastic pollution is becoming a serious challenge for crop productivity, human health, and ecosystem sustainability [1]. Plastics are an essential component of daily life; however, they are a concerning source of pollution in many ecosystems [2]. Plastic production and use have been steadily increasing worldwide, with global production reaching 460 million tons in 2019 and projected to quadruple by the end of 2060 [3]. Globally, around 80% of plastic produced for various purposes is released into the environment, where it can persist for several decades [4]. Plastic debris in the environment is converted into microplastics (<5 mm) and nanoplastics (<1 µm) through various processes, including biodegradation, ultraviolet radiation, and physical abrasion [5]. Polyethylene nanoplastics (NPs) reduce nutrient availability and soil bulk density, and compromise soil structure [6, 7]. They also alter soil pH and nutrient cycling, and all these processes directly influence the soil enzymatic and microbial activities [8]. Nanoplastics have strong hydrophobicity and surface areas, making them essential carriers of other pollutants [7]. The toxic substances present in NPs further augment their toxicity, thereby reducing crop growth [9]. They are now considered novel pollutants with a significant ability to enter the food chain and pose profound health implications. They also harm plants by increasing oxidative damage, reducing nutrient uptake, and disrupting root growth [10]. Furthermore, they damage cellular structures and the photosynthetic apparatus, reducing photosynthetic efficiency and thereby negatively affecting seedling establishment [11, 12].

In addition to NPs, heavy metal (HM) pollution is a serious challenge for humans and plants [13]. Among HMs, lead (Pb) is a highly toxic metal owing to its persistence and unique properties [14]. The concentration of Pb in agricultural lands has significantly increased due to anthropogenic activities [15]. Lead toxicity severely reduces plant growth by decreasing photosynthetic efficiency and transpiration and increasing ROS (reactive oxygen species) production [16, 17]. Lead disrupts electron transport, membrane integrity, and enzyme activity, leading to oxidative damage and subsequently impaired germination and seedling growth [18, 19]. It also damages the photosynthetic apparatus, decreases chlorophyll synthesis, and decouples the phosphorylation process, leading to a reduction in plant growth [20]. NPs have strong hydrophobicity and surface areas, making them essential carriers of other pollutants [11]. They absorb toxic metals, increasing their uptake and availability, which causes more poisonous impacts [11]. Recently, it was discovered that NPs and Pb inhibited germination, seedling growth, and biomass production, and enhanced antioxidant activity in wheat (*Triticum aestivum* L.) grown under hydroponic conditions [12]. Thus, it is critical to find ways to mitigate the adverse impacts of combined MPs and HMs on plants.

Globally, different techniques, including biochar, microbes, and hormone application, are used to mitigate the adverse impacts of HMs and NPs [11, 21, 22]. Recently, researchers also found that application of endophytic fungi (EF) is a rapid and cost‐effective technique to enhance crop productivity and stress tolerance [22]. Filamentous fungi (belonging to the genera *Trichoderma*, *Metarhizium*, and *Beauveria*) have shown metal‐detoxifying and growth‐promoting traits, supporting their broader applicability in phytoremediation and stress alleviation [22, 24]. Notably, fungi within the genus *Metarhizium* exhibit significant potential in heavy metal detoxification and plant growth promotion. *Metarhizium robertsii* has been applied for the bioremediation of mercury‐polluted soil and water through enzymatic detoxification mechanisms involving a methylmercury demethylase and a mercury ion reductase [25]. *M. robertsii* not only reduces metal accumulation in plants but also enhances plant growth under mercury stress, highlighting their dual role as bio‐remediators and plant symbionts [25]. Similarly, *Metarhizium anisopliae* (Metschn.) Sorokin, a well‐known insect pathogen and endophytic fungus [26], has also shown growth‐promoting effects by mitigating the impacts of abiotic stresses to enhance crop growth [27, 28]. Rice (*Oryza sativa* L.) is a staple food for over 3.5 billion people; nevertheless, toxic metals and MPs pose serious threats to rice productivity and food security. Heavy metals and NPs enter paddy soils through fertilizers, pesticides, sewage sludge, and atmospheric deposition, adversely affecting rice growth [29]. Although the individual toxic effects of MPs and Pb on plants are well documented, there is limited information on their interactive toxicity on rice and the potential role of endophytic fungi in mitigating these effects. Additionally, the interactive effects of NPs and Pb on rice physiology, transcriptomic and metabolic processes, and soil ecosystems remain poorly understood.

We propose that, in addition to the individual stress of Pb and NP, the co‐stress of lead Pb and NPs is attributable to a “Trojan Horse” mechanism, wherein NPs act as vectors facilitating the uptake and translocation of Pb into plant tissues. Furthermore, we hypothesize that inoculating seeds with the fungus *M. anisopliae* (SM) will mitigate this combined toxicity through a comprehensive “intercept‐and‐defend” strategy. Specifically, we posit that SM will (1) sequester Pb and Pb‐NP complexes at the root‐soil interface via biosorption, thereby reducing contaminant uptake; (2) confer protection by systematically enhancing the plant's antioxidant, detoxification, and photosynthetic pathways to restore cellular homeostasis; and (3) enhance plant health by enriching the rhizosphere with beneficial microorganisms. Hence, this study was undertaken to evaluate the individual and synergistic phytotoxic effects of Pb and NPs on rice and to assess their possible mitigation through *M. anisopliae* seed inoculation. The main objectives of this study were (1) to observe the phytotoxic effects of Pb and NPs (individual or combined) on growth parameters, antioxidant responses, hormone synthesis, transcriptome and metabolite profiles of rice; (2) to determine how *M. anisopliae* treatment improved different growth parameters, harmone synthesis and reduced antioxidant responses by inducing changes to transcriptome and metabolite profiles of rice; and (3) to elucidate whether *M. anisopliae* application also impacted the soil bacterial communities involved in toxicity alleviation. By integrating physiological traits with transcriptomic ‐metabolomic signatures and rhizosphere profiling, this study provides a systems‐level framework for fungal mitigation of emergent co‐contaminant stress supporting sustainable rice production under pollutant stress.

## 2 Experimental Methods

### 2.1 Soil, Lead, and Nanoplastic

The soil used for all the pot experiments (pH: 5.5; electrical conductivity: 0.1–0.5 mS·cm^−1^; organic matter content > 45%; bulk density 320–350 g·L^−1^; total nutrient content 2–4%; and moisture content < 30%) was a mixture of peat, vermiculite, and yellow clay. It was purchased from Guangzhou Shengsheng Agricultural Co., Ltd. The soil was sterilized at 121°C for 2 h, then air‐dried before use.

Lead nitrate (PbNO_3_), 0.2 mol L^−1^, used in this study for Pb contamination of soil, was supplied by Shenzhen Bidai Environmental Protection Technology Co., Ltd., China.

Polyethylene nanoplastic (NP) used in this study was obtained from Huabang Plastic Trading Co., Ltd., Dongguan, China. The average particle size of the NPs used was 100 nm, and they contained zinc stearate as a thermal stabilizer. Their surface morphology, examined by atomic force microscopy (AFM), revealed approximately spherical particles (Figure S1A). Zeta potential (surface charge) of NP was analyzed by using the Zetasizer Nano ZS90 (Malvern, UK). The zeta potential of NP was −6.83 mV (Figure S1B).

### 2.2 Metarhzium anisopliae

Seven strains of *M. anisopliae* (SM010, Genbank accession No. PX747585; SM021, Genbank accession No. PX747586; SM028, Genbank accession No. PX747587; SM036, Genbank accession No. PX747588; SM046, Genbank accession No. PX747589; SM047, Genbank accession No. PX747590; and SM073, Genbank accession No. PX747591), which were initially isolated from soil and identified following Du et al. [30], deposited at the Engineering Research Center of Biological Control, South China Agricultural University (SCAU), Guangzhou, China, were used during this study.

*M. anisopliae* slant cultures stored at 4°C were aseptically transferred to PDA plates in a UV‐sterilized laminar flow hood. After sealing with parafilm, the plates were incubated for 10 days. After 10 days of growth, conidia from the plate surface were scraped into a 0.05% Tween‐80 solution and stirred magnetically for 20 min to prepare a conidial suspension. The resulting suspension was sieved through filter paper (Whatman no. 2; Science Kit and Boreal Laboratories, New York, NY, United States) and collected into sterile vials. Conidia were counted using a compound microscope and a hemocytometer (0.0625 m^2^; Fuchs‐Rosenthal Merck Euro Lab, Darmstadt, Germany) to calibrate a suspension of 1 × 10^8^ conidia/mL.

### 2.3 Rice Seed Treatment

The rice variety Qingxiangyou 19 was used in the study and was provided by Guangdong Xianmei Seed Industry. First, seeds were sterilized using 4% NaClO for 4 h, then with 75% ethanol for 5 min. Rice seeds (30 seeds each) were dipped in conidial suspensions of different *M. anisopliae* isolates for 4 h, whereas seeds dipped in 0.05% Tween‐80 served as the control. All the seeds were allowed to air‐dry for 1 h before sowing.

### 2.4 Preliminary Screening of *M. anisopliae* Strains

The present experiment was conducted at the Engineering Research Center of Biological Control, South China Agricultural University (SCAU), Guangzhou, China. In the initial screening phase, seven strains of *M. anisopliae* (SM010, SM021, SM028, SM036, SM046, SM047, and SM073) were evaluated for their plant growth‐promoting effects. The seeds were sown in plastic pots (13.5 cm × 12.5 cm × 17.5 cm) containing 100 g of pre‐sterilized air‐dried soil (soil properties mentioned in Table S1), with 50 mL of water added. Pots were placed in a room maintained at a controlled temperature of 26 ± 1°C (controlled by a temperature control unit). Seedlings were harvested after 20 days to measure various growth traits. The experiment was replicated thrice.

### 2.5 Pot Experiment Design and Stress Treatments

The soil was divided into four equal parts (200 g each) and mixed with different amendments to obtain the treatments, following Zhang et al. [31]. The first part of the soil was amended with a stock solution of PbNO_3_ to prepare lead concentrations of 100 mg/kg in the soil. The other part was amended with Polyethylene nanopalstic (NP) at a concentration of 2000 mg/kg soil. The third part of the soil was amended with a combination of PbNO3 and Polyethylene nanopalstic to obtain a final concentration of 100 mg/kg Pb+2000 mg/kg NP. The fourth part of the soil was left untreated. The amended soils were thoroughly mixed and left to equilibrate for 45 days. The physical properties of soils spiked with different treatments, as well as non‐spiked soil, at the end of 45 days of equilibration are shown in Table S1. After 45 days, 100 g of soil from each treatment group was individually filled into plastic pots (13.5 cm × 12.5 cm × 17.5 cm), and 50 mL of water was added to each pot. Finally, pots containing soil spiked with different treatments were divided into two subgroups (each subgroup comprising four pots from the four treatments mentioned above). Rice seeds (3 seeds/pot) soaked in conidial suspension (1 × 10^8^ conidia/mL) of *M. anisopliae* strain SM021, as described in Section 2.3, were sown into pots from one subgroup. In contrast, seeds soaked in 0.05% Tween‐80 were sown into pots from another subgroup. Finally, study contained eight treatments: CK (untreated seeds sown in non‐spiked soil); SM (seeds treated with *M. anisopliae* SM021 sown in non‐spiked soil); Pb (untreated seeds sown in soil spiked with Pb treatment); PbSM (seeds treated with *M. anisopliae* SM021 sown in soil spiked with Pb treatment); NP (untreated seeds sown in soil spiked with NP treatment); NPSM (seeds treated with *M. anisopliae* SM021 sown in soil spiked with NP treatment); PbNP (untreated seeds sown in soil spiked with combined Pb + NP treatment); and PbNPSM (seeds treated with *M. anisopliae* SM021 sown in soil spiked with combined Pb + NP treatment). Pots were placed in a room maintained at a controlled temperature of 26 ± 1°C (controlled by a temperature control unit). The whole experimental setup was replicated thrice. After 20 days of growth, rice seedlings from each group were collected. One subset was used to measure growth parameters (plant height, root length, fresh weight, and dry weight) and physiological indicators (chlorophyll content, antioxidant enzyme activities, oxidative stress markers, and phytohormone levels). Another subset was stored at −80°C for transcriptomic and metabolomic sequencing. All phenotypic, physiological, and omics analyses were performed with three biological replicates.

### 2.6 Measurement of Pb Concentration

Concentration of Pb was determined by weighing 1.0 g of air‐dried soil sample, followed by digestion using a mixed acid procedure (HNO_3_‐HCl‐HF‐HClO_4_). The digested solution was analyzed by inductively coupled plasma mass spectrometry (ICP‑MS). Plant Pb concentration was measured by weighing 1.0 g of oven‐dried plant tissue (roots or leaves). Samples were digested with concentrated HNO_3_ in a microwave digestion system. The clear digestates were then analyzed for Pb content using ICP‑MS [31].

### 2.7 Measurement of Growth Traits

Plants were carefully harvested, and plant height and root length were measured with a measuring tape. The plants were weighed to measure fresh weights, then oven‐dried (60°C) for 72 h, and weighed again to measure dry weight.

### 2.8 Measurement of Antioxidant Activities and Hormone Concentration

For all physiological and biochemical measurements, fresh rice leaf samples (0.1 g) were collected and frozen using liquid nitrogen. The rice samples were homogenized in an ice bath, and the supernatant was collected. All antioxidant enzyme activities and phytohormone concentrations were determined using the following commercial assay kits (supplied by Beijing Boxbio Science & Technology Co., Ltd. and Jiangsu Meimian Industrial Co., Ltd) according to the manufacturer's instructions: Superoxide Dismutase (SOD) Activity Assay Kit (Catalog Number AKAO001M‐100S); Catalase (CAT) Activity Assay Kit (Catalog Number AKAO003‐2M); Peroxidase (POD) Activity Assay Kit (Catalog Number AKAO005M); Malondialdehyde (MDA) Content Assay Kit (Catalog Number AKFA013M); Hydrogen Peroxide (H_2_O_2_) Content Assay Kit (Catalog Number AKAO009M); Plant Chlorophyll Content Assay Kit (Catalog Number AKPL003M); Plant Gibberellin (GA) ELISA Kit (Catalog Number MM‐0125O1); Plant Auxin (IAA) ELISA Kit (Catalog Number MM‐0953O1); Plant Jasmonic Acid (JA) ELISA Kit (Catalog Number MM‐0887O1); and Plant Salicylic Acid (SA) ELISA Kit (Catalog Number MM‐33722O2).

### 2.9 Microbiome Analysis

For microbial analysis, rhizosphere soil samples were collected from each treatment group, with three biological replicates per group. Approximately 3 g of soil from each replicate was sampled, immediately flash‑frozen in liquid nitrogen. Soil DNA was extracted using the PowerSoil Kit (MoBio). The V3–V4 region of 16S rRNA was amplified with 338F/806R primers and sequenced on Illumina NovaSeq. After quality filtering (DADA2), the SILVA database was used to classify the amplicon sequence variants (ASVs). ASV‐level alpha diversity indices, such as the Chao richness estimator, Shannon diversity index, and Simpson index, were calculated using the ASV table in QIIME2 and visualized as box plots. Beta diversity analysis was performed to investigate the structural variation of microbial communities across samples using Jaccard metrics, and the results were visualized via principal coordinate analysis (PCoA), nonmetric multidimensional scaling (NMDS), and unweighted pair‐group method with arithmetic means (UPGMA) hierarchical clustering. Principal component analysis (PCA) was also conducted based on the genus‐level compositional profiles. Microbial functions were predicted using PICRUSt2 (Phylogenetic investigation of communities by reconstruction of unobserved states) based on the MetaCyc (https://metacyc.org/) and KEGG (https://www.kegg.jp/) databases. All analyses were conducted on the Majorbio Cloud platform (https://cloud.majorbio.com).

### 2.10 Transcriptomic Analysis and Metabolomic Profiling

Fresh rice leaves were frozen in liquid N, and RNA was extracted using Trizol reagent. Nanodrop2000 and agarose gel electrophoresis verified its quality. Poly(A)‐enriched mRNA libraries were prepared and sequenced on the NovaSeq X Plus platform (Illumina). After quality control using fastp, clean reads were aligned to the rice reference genome (IRGSP‐1.0) using HISAT2. Moreover, DESeq2 (FDR < 0.05, |log2FC| ≥ 1) was used to identify differentially expressed genes (DEGs). Genes with similar expression patterns often exhibit identical functions. The R toolkit pheatMap software was used for cluster analysis of DEGs and experimental conditions. Functional analysis of these DEGs was conducted using the Kyoto Encyclopedia of Genes and Genomes.

For metabolomic analysis, frozen leaves were extracted with methanol:water (4:1) containing 0.02 mg/mL L‐2‐chlorophenylalanine as the internal standard. After homogenization and centrifugation, the supernatants were analyzed using a UHPLC‐Q Exactive HF‐X system (Thermo Scientific). Raw data were processed using Progenesis QI, with metabolites identified against HMDB and Metlin databases. Multivariate analysis (PCA/OPLS‐DA) was performed using the ropls package to assess the significance (*p*‐value) of the metabolites. Metabolites that satisfied the following criteria were considered differentially abundant: VIP>1, *p* value<0.05, and fold change (FC)≥2.0 or FC≤0.5. Volcano plots were used to discern and select metabolites of significance. Heatmaps and Venn diagrams were used to visualize changes in metabolites across distinct groupings. The roles and activities of these metabolites and metabolic pathways were investigated via the KEGG database. The identification of enhanced metabolic pathways was conducted based on the differential abundance of metabolites. Metabolic pathways were deemed enriched when the ratio x/n>y/N was satisfied. Metabolic pathways were deemed statistically substantially enriched when their *p* values were less than 0.05.

Differentially accumulated metabolites (DAMs) and DEGs with a Pearson correlation coefficient greater than 0.8 were used to generate a nine‐quadrant plot for systematic comparison of dynamic changes in DAMs and DEGs across stages. Integrated transcriptomic and metabolomic analyses were conducted on the Majorbio Cloud platform. The hypergeometric distribution algorithm was applied to identify significantly enriched pathways in the gene and metabolite sets, respectively, to explore relationships between metabolites and genes involved in the same biological processes.

### 2.11 Statistical Analysis

Statistical analysis was performed using SPSS software (v29.0). All plant growth and physiological, biochemical data were subjected to one‐way analysis of variance (ANOVA) (*p* < 0.05). All data followed a normal distribution before performing analysis of variance (ANOVA). Furthermore, Duncan's multiple range test was applied for post hoc comparisons. Data are expressed as mean ± SE. All graphical representations were created using GraphPad Prism 10.

## 3 Results

### 3.1 Preliminary Screening of Fungal Strains

Data on improvement in rice growth induced by seed treatment with seven different *M. anisopliae* strains are shown in Figure S2. The results indicated that different fungal strains significantly affected rice growth traits. Overall, fungal strain SM021 performed appreciably well as compared to other fungal strains by inducing a significant increase in all the growth traits. Notably, the highest average plant height (33.83 cm), average root length (11.00 cm), average plant fresh weight (328.33 mg), and average dry weight (96.00 mg) were observed in the SM021 treatment group. These results proved the feasibility of using the *M. anisopliae* SM021 strain for subsequent studies.

### 3.2 Effects of *M. anisopliae* Inoculation on Pb Accumulation and Translocation

The results indicated that seed inoculation with *M. anisopliae* reduced lead (Pb) accumulation in rice tissues and altered its translocation ratios (Table 1). Compared to the Pb‑alone treatment, inoculation (PbSM) significantly lowered the soil‑to‑root transfer factor by 35.7% (from 1.1358 to 0.7308). Similarly, under combined Pb+NP stress, fungal inoculation (PbNPSM) decreased the soil‑to‑root transfer coefficient by 34.4% relative to the PbNP group (from 1.2308 to 0.8077). In rice, Pb is predominantly retained in the roots, with only a small fraction translocated to the leaves. These reductions indicate that *M. anisopliae* effectively restricted Pb uptake from soil into roots.

**TABLE 1:** Pb transfer coefficients among components of soil‐roots‐leaves.

Treatments	Soil—roots	Roots—leaves
Pb	1.1358	0.0276
PbSM	0.7308	0.0142
PbNP	1.2308	0.0285
PbNPSM	0.8077	0.0158
CK	0.3360	0.0116
SM	0.3408	0.0113
NP	0.3641	0.0135
NPSM	0.3507	0.0121

### 3.3 Effects of *M. anisopliae* Inoculation on Growth Traits and Chlorophyll Contents of Rice Under Pb and NP Contaminated Soil

**FIGURE 1:**
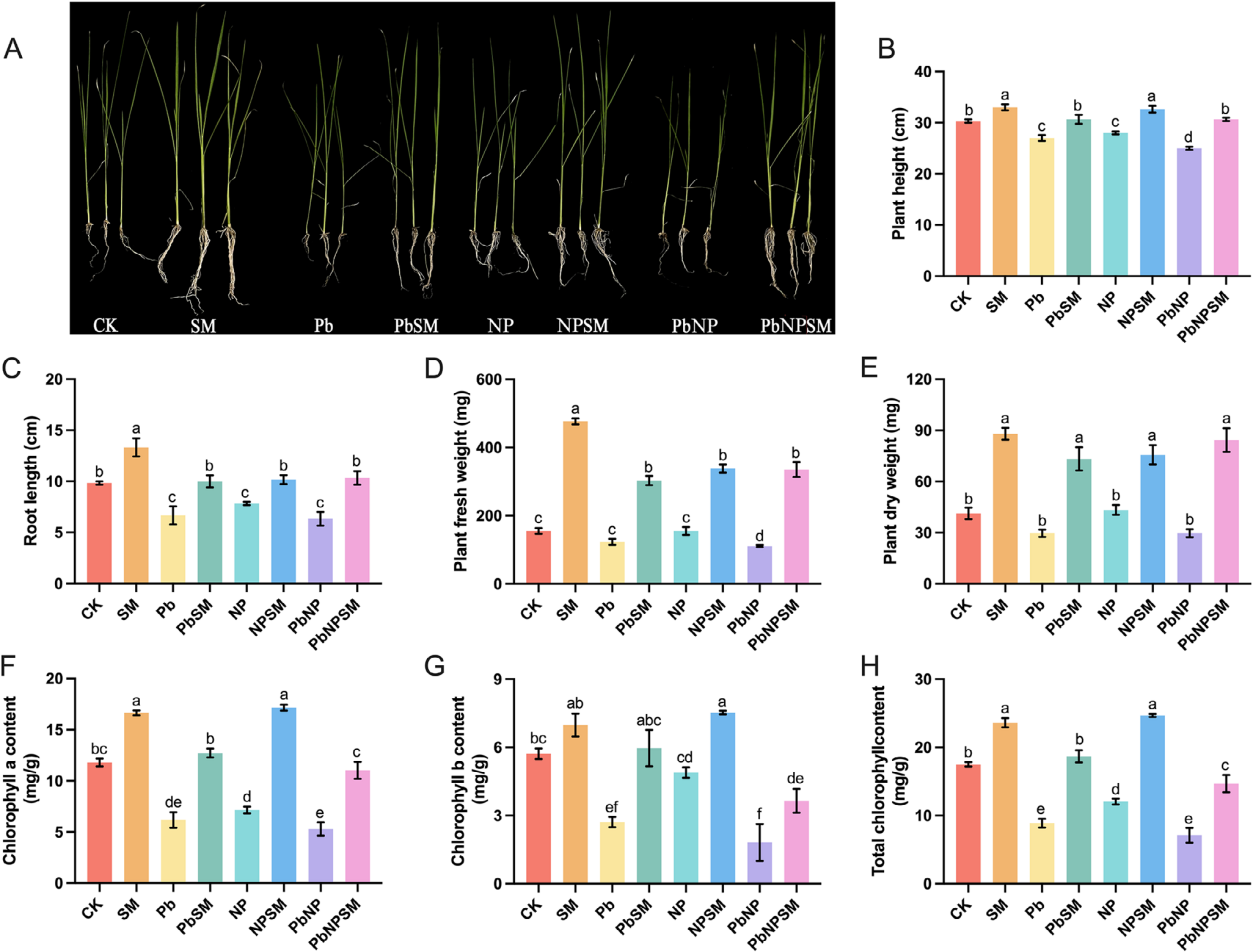
Effect of *M. anisopliae* (SM021) inoculation on the morphological and physiological traits of rice plants grown under lead (Pb), polyethylene (NP), and combined Pb and NP‐contaminated soil. (A) Morphology of shoots and roots; (B) plant height; (C) root length; (D) plant fresh weight; (E) plant dry weight; (F) chlorophyll a content; (G) chlorophyll b content; and (H) total chlorophyll content. Data represent mean ± SE from three replicates per treatment. Different lowercase letters indicate statistically significant differences among treatments at *p* < 0.05. Note: CK (untreated seeds sown in non‐spiked soil); SM (seeds treated with M. anisopliae SM021 sown in non‐spiked soil); Pb (untreated seeds sown in soil spiked with Pb treatment); PbSM (seeds treated with M. anisopliae SM021 sown in soil spiked with Pb treatment); NP (untreated seeds sown in soil spiked with NP treatment); NPSM (seeds treated with M. anisopliae SM021 sown in soil spiked with NP treatment); PbNP (untreated seeds sown in soil spiked with combined Pb + NP treatment); and PbNPSM (seeds treated with M. anisopliae SM021 sown in soil spiked with combined Pb + NP treatment).

The results indicated that different treatments significantly impacted rice growth (Figure 1). Pb and NP significantly inhibited plant growth, with the most pronounced effects observed in the combined treatment (PbNP), which decreased the plant height (PH), root length (RL), root fresh weight (RFW), and root dry weight (RDW) by 17.58%, 35.59%, 28.60% and 28.23% respectively, as compared to the control. However, seed treatment with *M. anisopliae* significantly offset the negative impacts of Pb and NP and substantially enhanced the rice growth. Notably, *M. anisopliae* enhanced the PH, RL, RFW, and RDW 22.67%, 63.16%, 203.01% and 184.27% respectively, as compared to PbNP (Figure 1). Chlorophyll synthesis was also significantly decreased in Pb‐ and NP‐polluted soil. The PbNP group caused the most severe reduction in chlorophyll a, b, and total chlorophyll content, decreasing them by 55.09%, 68.30%, and 59.40%, respectively.

Conversely, the application of SM021 significantly increased the synthesis of all chlorophyll fractions under all stress conditions (Pb, NP, and PbNP). Specifically, compared to the PbNP group, the PbNPSM group exhibited remarkable increases of 108.15%, 101.41%, and 106.43% in chlorophyll a, b, and total chlorophyll content, respectively. These results demonstrate that SM021 can effectively alleviate the toxic effects caused by individual and combined Pb and NP stress.

### 3.4 Effects of *M. anisopliae* Inoculation on Antioxidant Enzymes, Oxidative Stress Markers, and Endogenous Hormones Synthesis under Individual or Combined Pb and NP Stress

**FIGURE 2:**
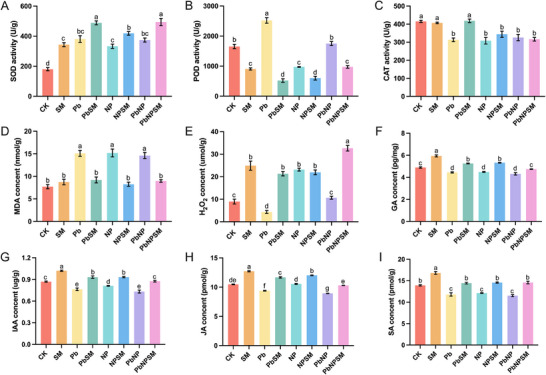
Effect of *M. anisopliae* (SM021) inoculation on antioxidant enzyme activities, oxidative stress markers, and endogenous hormone synthesis in rice plants grown under lead (Pb), polyethylene (NP), and combined Pb and NP‐contaminated soil. (A) Superoxide dismutase (SOD); (B) peroxidase (POD); (C) catalase (CAT); (D) malondialdehyde (MDA); (E) hydrogen peroxide (H_2_O_2_); (F) gibberellin (GA); (G) indole‐3‐acetic acid (IAA); (H) jasmonic acid (JA); and (I) salicylic acid (SA). Data represent mean ± SE from three replicates per treatment. n = 3. Different lowercase letters indicate statistically significant differences among treatments at *p* < 0.05.

Different treatments significantly increased antioxidant activity and produced oxidative stress markers in the Pb, NP, and PbNP groups (Figure 2). The SOD activities of rice seedlings from *M. anisopliae*‐treated or untreated groups in response to individual or combined stress of Pb and NP were significantly different from each other, whereas SOD activities observed for all treatments were significantly higher than the control. The PbSM, NPSM, and PbNPSM groups all significantly increased SOD activities, establishing a balanced antioxidant to counter Pb and NP toxicity (Figure 2A). The POD activities of rice seedlings from *M. anisopliae*‐treated or untreated groups in response to individual or combined stress of Pb and NP were significantly different from each other, whereas POD activities observed for all treatments were significantly lower than the control (except for Pb treatment). The PbSM, NPSM, and PbNPSM groups all showed substantially lower POD than the Pb, NP, and NPSM groups (Figure 2B). The CAT activities of rice seedlings from the SM and PbSM treatment groups were similar to those of the control. In contrast, the CAT activities of rice seedlings from the remaining treatments were significantly lower than those of the control (Figure 2B).

The changes in H_2_O_2_ content are as follows: compared to the CK group, the Pb group decreased by 51.32%, the NP group increased by 157.82%, and the PbNP group increased by 18.79%. Furthermore, Pb, NP, and PbNP increased MDA production by 96.09%, 97.21%, and 89.81%, respectively (Figure 2). Notably, inoculation with the SM021 strain significantly reduced MDA levels under all stress conditions: MDA decreased by 39.03% in PbSM compared to Pb, by 45.61% in NPSM compared to NP, and by 38.49% in PbNPSM compared to PbNP. In contrast, H_2_O_2_ content showed a distinct response to fungal inoculation: it increased by 388.14% in PbSM compared to Pb, decreased by 5.23% in NPSM compared to NP, and increased by 206.17% in PbNPSM compared to PbNP. Different stress treatments manifested the antioxidant activities.

It was observed that stress treatments such as Pb and NP, and particularly PbNP, significantly inhibited the synthesis of GA, IAA, JA, and SA. Notably, PbNP significantly inhibited GA, IAA, JA, and SA synthesis by 11.78%, 15.79%, 14.69%, and 17.23%, respectively, compared to the control. Fungal treatment significantly increased the levels of all measured phytohormones. It restored phytohormone synthesis near the CK groups, thereby conferring resistance in rice plants to Pb, NP, and combined PbNP toxicity.

### 3.5 Effects of *M. anisopliae* Inoculation on Soil Bacterial Community Composition Under Pb and NP Contaminated Soil

**FIGURE 3:**
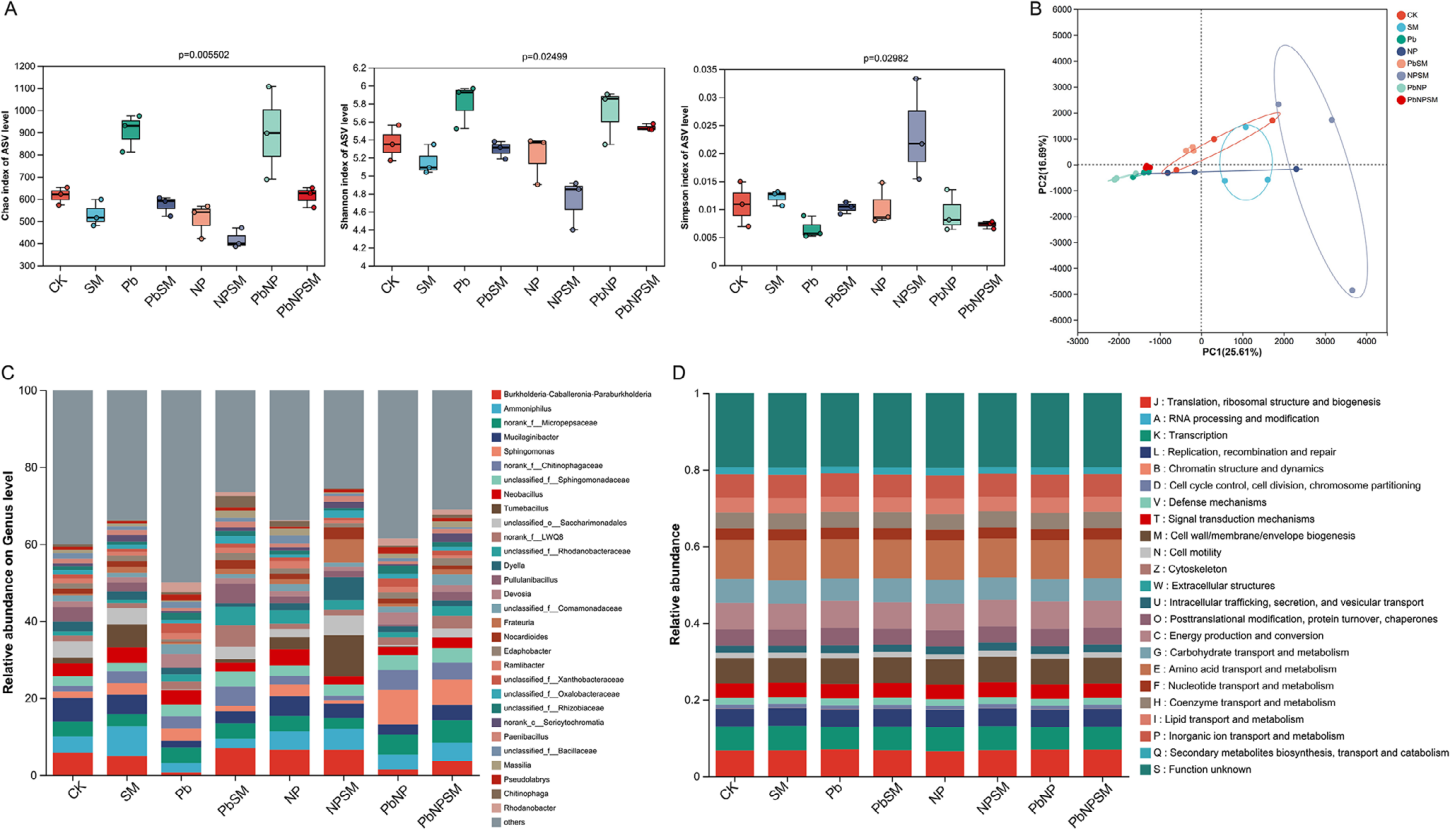
Effect of *M. anisopliae* SM021 inoculation on microbial responses in rice rhizosphere soil under Pb, NP, and PbNP stress. The panel presents (A) Chao index, Shanoon index, and Simpson index of ASV level, (B) multivariate separation showing treatment clustering, (C) relative abundance of key microbial taxa in the rhizosphere soil, and (D) pathway‐level shifts in major functional groups.

Different treatments changed the composition and structure of the microbial community in the rhizosphere soil of rice (Figure 3A). Principal coordinates analysis (PCoA) based on Bray–Curtis distances further illustrated structural differences across groups (Figure 3B). The results indicated that different treatments significantly altered the structure of the rhizosphere core microbiota (Figure 3C). Stress treatments, including Pb, NP, and PbNP, significantly reduced the abundance of specific bacterial taxa. For instance, the abundance of beneficial bacteria with roles in plant growth promotion and organic pollutant degradation (*Burkholderia*‐*Caballeronia*‐*Paraburkholderia*) was reduced to 0.72% under Pb and 1.50% under PbNP treatments, respectively, markedly lower than in the CK group (5.73%). Likewise, the abundance of the polysaccharide‐degrading bacterium *Mucilaginibacter* also decreased to 1.67% under Pb stress compared to CK (6.06%). This indicated that heavy metal stress has a strong inhibitory effect on beneficial rhizosphere microorganisms. SM021 fungal treatment effectively reversed the above‐mentioned inhibitory trend and enriched the key beneficial bacterial genera. Under Pb stress and treated with *M. anisopliae* SM021, increased *Burkholderia*‐*Caballeronia*‐*Paraburkholderia* abundance to 7.07% and *Chitinophaga* abundance to 3.02%. In NP treatment, SM021 enhanced *Tumebacillus* to 10.73%. PbNP stress led to abnormal enrichment of *Sphingomonas* (8.96%) and *norank*_*f*__*Micropepsaceae* (5.27%), which might be a stress response of the microbial community to extreme stress. However, SM021 treatment of PbNP stress maintained a high *Sphingomonas* (6.59%) and an abundance of *Burkholderia*‐*Caballeronia*‐*Paraburkholderia* (3.66%) that recovered to more than twice that of the PbNP group (1.50%) (Table S2).

Figure 3D shows the functional prediction analysis of microorganisms. The main enriched pathways were translation, ribosomal structure and biogenesis, RNA processing and modification, transcription, and replication. Furthermore, cell divisions, signal transduction, defense mechanisms, cytoskeleton, intercellular trafficking, protein turnover, energy production, carbohydrates, lipids, amino acids, and the transport and metabolism of secondary metabolites were the main enriched pathways (Figure 3D). Furthermore, LEfSe analysis was performed to identify microbial biomarkers with significant differences in relative abundance (Figure S3). The results indicated that, compared with the CK vs SM group, the differentially abundant groups in the SM group were *Alicyclobacillaceae*, *Alicyclobacillales*, *Tumebacillus*, *Chitinophagaceae*, *Sphingomonas*, *Pandoraea*, *Chloroplast*, *Cyanobacteriia*, *Fibrisoma*, and (Figure S3A). In the CK vs Pb group, the abundance groups in Pb were *Hyphomicrobiales*, *Gemmatimonadota*, *Gemmatimonadales*, *Gemmatimonadia*, *Chitinophagales*, *Xanthobacteraceae*, *Gemmatimonadaceae*, *Chitinophagaceae*, *Rhodanobacter*, and *Devosia* (Figure S3B). While in the CK vs PbSM group, the abundant bacteria in PbSM were *Chitinophagaceae*, *Gammaproteobacteria*, *Chitinophagales*, *Lysobacterales*, *LWQ8*, *Rhodanobacteraceae*, and *Chitinophagacea, Chitinophaga*, and *Oxalobacteraceae* (Figure S3C). Moreover, in the CK vs NP group, the abundant bacteria in the NP groups were *Sphingomonas*, *Frateuria*, *Acetobacteraceae*, *Acetobacterales*, *Xanthobacteraceae*, *Arachidicoccus*, *Babeliae*, *Babeliales*, and *Dependentiae*, *Vermiphilaceae* (Figure S3D). In contrast, in the CK vs NPSM group, the abundant groups in NPSM were *Lysobacterales*, *Rhodanobacteraceae*, *Alicyclobacillales*, *Tumebacillus*, *Alicyclobacillaceae*, *Gammaproteobacteria*, and *Actinomycetota*, *Actinobacteria*, *Legionella*, *Legionellaceae* (Figure S3E). Lastly, in the CK vs PbNP group, the most abundant bacteria in PbNP were *Alphaproteobacteria*, *Pseudomonadota*, *Sphingomonadaceae*, *Sphingomonadales*, *Sphingomonas*, *Hyphomicrobiales*, *Chitinophagaceae*, *Chitinophagales*, and *Xanthobacteraceae* (Figure S3F). In contrast, in CK vs PbNPSM group, the abundant bacteria in PbNPSM group were *Alphaproteobacteria*, *Pseudomonadota*, *Sphingomonadaceae*, *Sphingomonadales*, *Sphingomonas*, *Chitinophagaceae*, and *Chiti nophagales*, *Chitinophagaceae*, and *LWQ8* (Figure S3G).

### 3.6 Effects of *M. anisopliae* Inoculation on Differential Gene Expression (DEGs) Under Pb and NP Contaminated Soil

**FIGURE 4:**
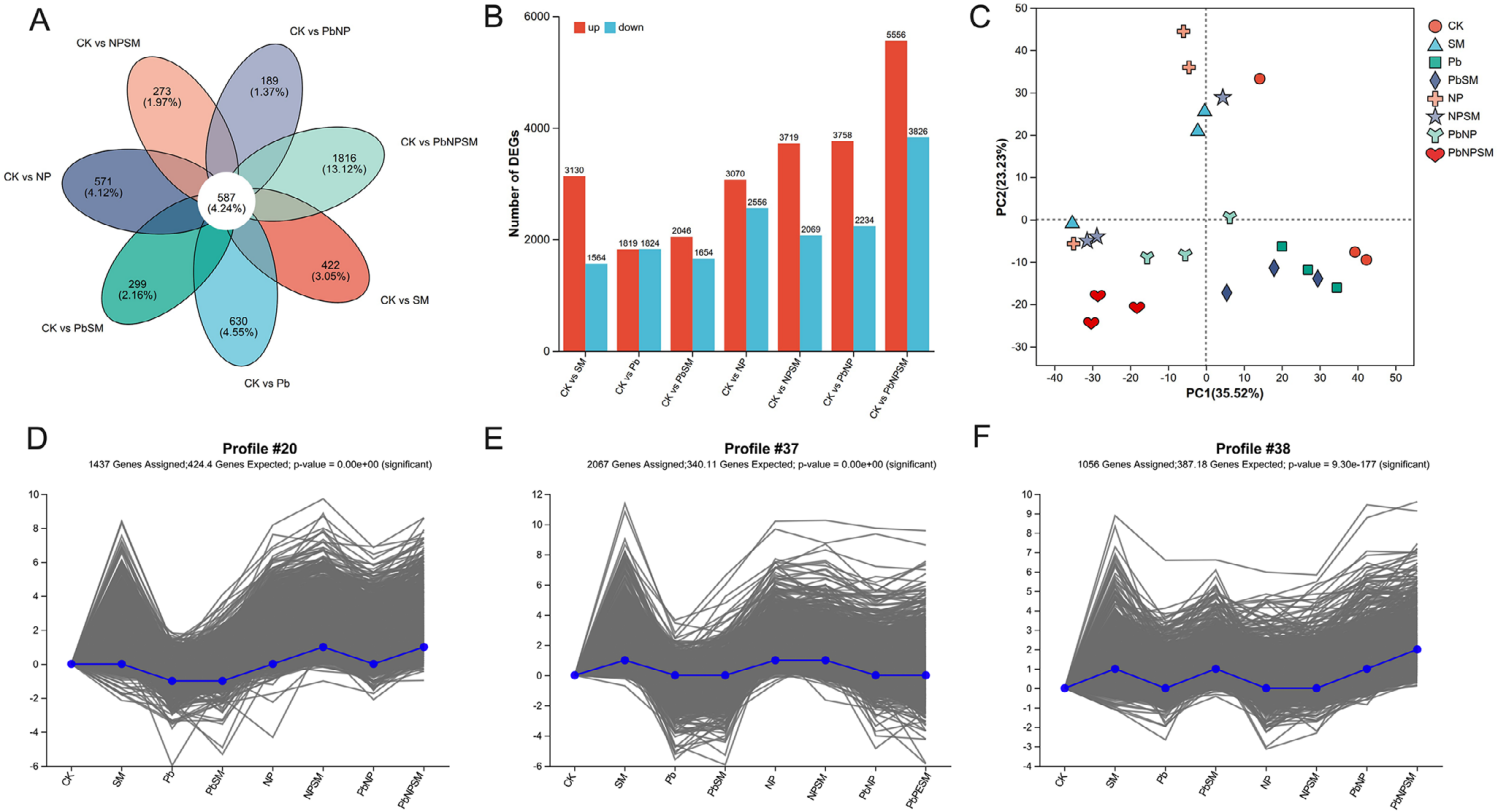
Effects of *M. anisopliae* SM021 inoculation on gene expression in rice under Pb, NP, and PbNP stress. The panel presents (A) venn diagram of overlapping DEGs among treatments, (B) up‐/down‐regulation patterns of DEGs, (C) principal component analysis (PCA) of samples, and (D–F) trend analysis of up‐/down‐regulated DEGs under different treatments (*p*<0.05, fold change>2.0).

A transcriptome analysis was performed on eight experimental groups. A Venn diagram was generated for each comparison group (Figure 4A). The number of up‐ and down‐regulated differentially expressed genes (DEGs) in each comparison group (all compared to CK) is shown in Figure 4B. Notably, compared to CK, the SM group had 3130 up‐regulated and 1564 down‐regulated genes; the Pb group had 1819 up‐regulated and 1824 down‐regulated genes. Moreover, the PbSM group had 2046 up‐regulated and 1654 down‐regulated genes; the NP group had 3030 up‐regulated and 2556 down‐regulated genes, and the NPSM group had 3719 up‐regulated and 2069 down‐regulated genes. Additionally, the PbNP group had 3758 up‐regulated and 2234 down‐regulated genes, and the PbNPSM group had 5556 up‐regulated and 3826 down‐regulated genes. PCA revealed significant variation among groups in gene expression (Figure 4C). To more precisely identify the core gene set regulated by SM, we performed time‐series expression pattern clustering on all DEGs using the Short Time‐series Expression Miner (STEM). The identified DEGs were categorized into 12 expression patterns (Figure S4), and three profiles with the most significant expression characteristics—Profile20, 37, and 38—were selected (Figure 4D–F). These genes were up‐ or down‐regulated under Pb, NP, and combined stress treatments, but exhibited opposite regulatory trends under the corresponding fungal treatment. Different treatments also significantly affected the pathways enriched for DEGs. The detailed results for different pathways enriched for DEGs are presented in (Figure S5).

### 3.7 Effects of *M. anisopliae* Inoculation on Expression of Different Metabolites Under Pb and NP Contaminated Soil

**FIGURE 5:**
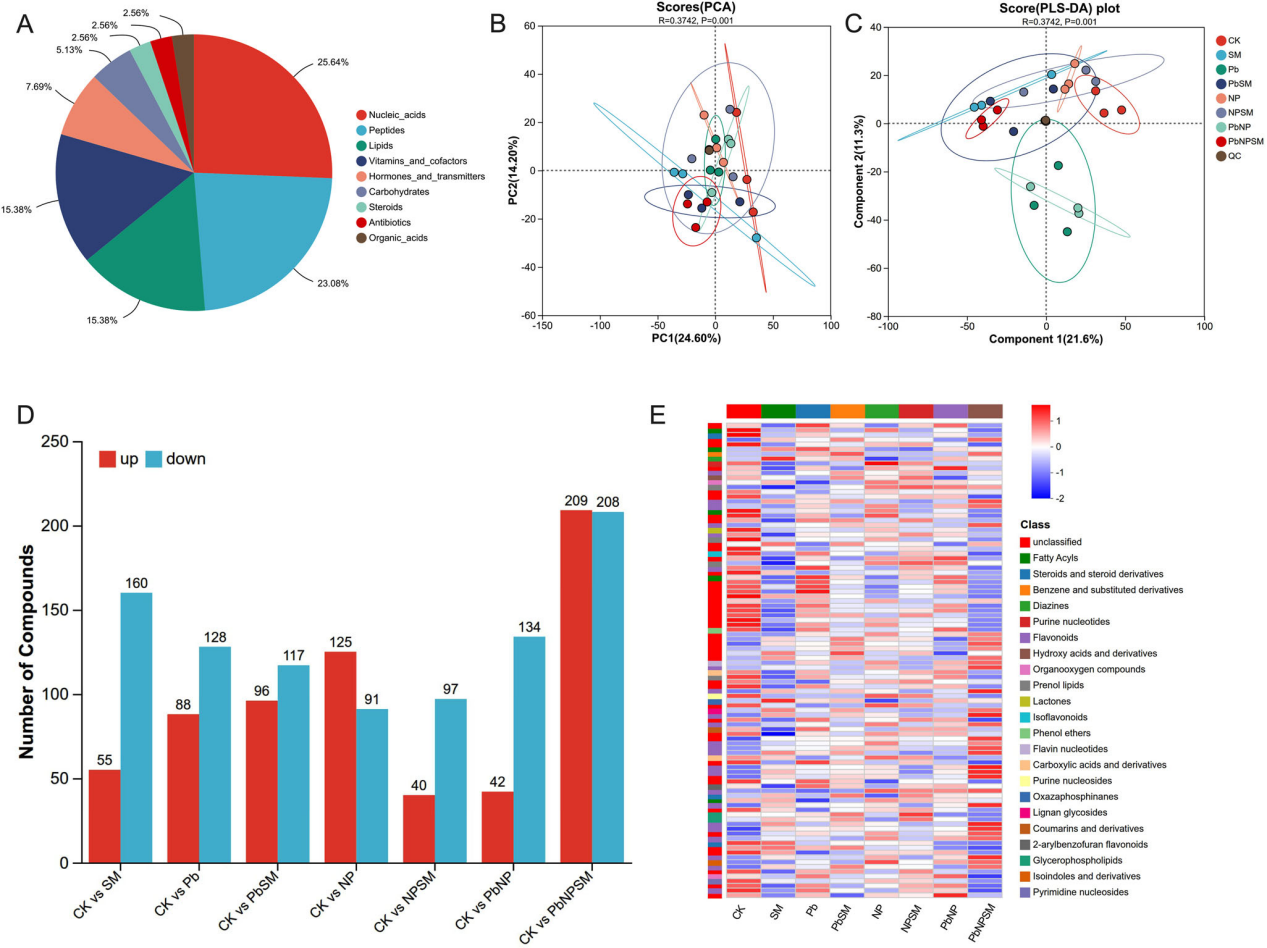
Effects of *M. anisopliae* SM021 inoculation on metabolites expression in rice under Pb, NP, and PbNP stress. The panel presents (A) classification and quantity of DAMs, (B) PCA of samples, (C) PLS‐DA of samples, (D) up‐/down‐regulation patterns of DAMs, and (E) heatmap analysis of DAMs across groups. [Correction added on 2 March 2026 after online publication: figure 5 is updated in this version.]

A total of nine chemical classes of metabolites were identified in the metabolome (Figure 5A). The most predominant class was nucleic acids (25.64%), followed by other categories including peptides (23.08%), lipids (15.38%), vitamins and cofactors (15.38%), hormones and transmitters (7.69%), carbohydrates (5.13%), steroids (2.56%), antibiotics (2.56%), and organic acids (2.56%). Principal component analysis (PCA) of the samples is shown in Figure 5B, while partial least squares‐discriminant analysis (PLS‐DA) is presented in Figure 5C. The upregulation and down‐regulation of differentially expressed metabolites in each comparison group (all compared to CK) are illustrated in Figure 5D. Compared to CK, the SM group showed upregulation of 55 metabolites and downregulation of 160 metabolites. Compared to CK, the Pb group showed upregulation of 88 metabolites and downregulation of 128 metabolites. Compared to CK, the PbSM group showed upregulation of 96 metabolites and downregulation of 117 metabolites. The NP group showed upregulation of 125 metabolites and downregulation of 91 metabolites than control. Further NPSM group showed upregulation of 40 metabolites and downregulation of 97 metabolites than control and PbNP group showed upregulation of 42 metabolites and downregulation of 134 metabolites than control. Moreover, PbNPSM group showed upregulation of 209 metabolites and downregulation of 208 metabolites than control growth.

The differentially expressed metabolites were primarily enriched in fatty acyls, steroids, and steroid derivatives, benzene and substituted derivatives, diazines, purine nucleotides, flavonoids, hydroxy acids and derivatives, organooxygen compounds, prenol lipids, lactones, isoflavonoids, phenol ethers, flavin nucleotides, carboxylic acids and derivatives, purine nucleosides, oxazaphosphinanes, lignan glycosides, coumarins and derivatives, 2‐arylbenzofuran flavonoids, glycerophospholipids, isoindoles and derivativespyrimidine nucleosides (Figure 5E). The detailed results for different DAMs up‐ and down‐regulated across treatment groups are presented in the supplementary material section (Figure S6). Furthermore, details of the KEGG enrichment analysis of DAMs under different treatments are presented in Figure S7.

### 3.8 Integrated Analysis of Transcriptome and Metabolome

**FIGURE 6:**
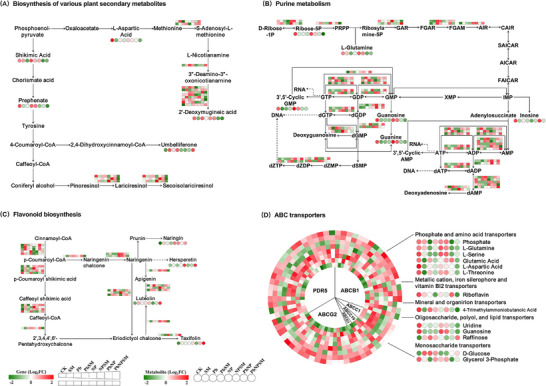
Effects of *M. anisopliae* SM021 inoculation on major metabolic pathways in rice under Pb, NP, and PbNP stress. (A) biosynthesis of various plant secondary metabolites, (B) Purine metabolism, (C) Flavonoid biosynthesis, and (D) ABC transporters. Circles represent different metabolites, boxs represent different genes. Color intensity shows up‐/down‐regulation of DEGs/DAMs (red = upregulated, green = downregulated).

To investigate the complex interaction between DEGs and DAMs, a comprehensive co‐expression network analysis was conducted. These analyses employed strict thresholds (Pearson correlation coefficient > 0.8, *p* value < 0.05) to ensure statistical significance and biological relevance. The results showed that many DEGs exhibit a strong positive correlation with DAMs (Figure S8). KEGG enrichment analysis was performed to identify pathways co‐enriched with both DEGs and DAMs. The results showed that in CK vs SM group top 20 enriched pathways DEGs and DAMs co‐enriched in the same pathways in CK vs SM group were porphyrin, monobactam, glycine, serine, threonine, glutathione, galactose and histidine metabolisms and lysine, arginine, flavonoid, pantothenate, folate biosynthesis, beta‐alanine, alanine, aspartate, glutamate, proline, arginine, cysteine, purine, methionine metabolisms, ABC transporters and Aminoacyl‐tRNA biosynthesis (Figure S9A). In CK vs Pb group, 20 significantly enriched pathways were glycine, serine and threonine metabolism, pyrimidine metabolism, glycerophospholipid metabolism, oxidative phosphorylation photosynthesis, flavone and flavonol biosynthesis, fructose and mannose metabolism, flavonoid biosynthesis, glucosinolate biosynthesis, alpha‐linolenic acid metabolism, sulfur metabolism, one carbon pool by folate, folate biosynthesis, pentose and glucuronate interconversis, cysteine and methionine, purine metabolisms, diterpenoid biosynthesis, aminoacyl‐tRNA biosynthesis and ABC transporters (Figure S9B). In the CK vs PbSM group, the top 20 significantly enriched pathways were pentose phosphate pathway, glycine, serine, threonine, aldarate, ascorbate, amino sugars, fructose, mannose, alpha‐linolenic acid, cysteine, methionine, and purine metabolisms, and purine, stilbenoid, gingerol, valine, leucine, aminoacyl‐tRNA, isoleucine biosynthesis, and ABC transporter (Figure S9C). In CK vs NP group, top 20 significantly enriched pathways were alpha‐linolenic acid metabolism, flavone and flavonol biosynthesis, vitamin B6 metabolism, linoleic acid, tropane, cysteine, methionine, purine, lysine, aldarate, ascorbate, galactose, and glycerophospholipid metabolisms, flavonoid, carotenoid, piperidine, pyridine, mono‐bactam, secondary metabolites biosynthesis, ABC transporters and hormones signal transduction (Figure S9D). Moreover, In CK vs NPSM, top 20 significantly enriched pathways were One carbon pool by folate, pentose phosphate pathway, vitamin b6 metabolism, carotenoid biosynthesis, folate biosynthesis, glycine, serine and threonine metabolism, flavonoid biosynthesis, lipoic acid metabolism, monoterpenoid biosynthesis, carbon fixation in photosynthetic organisms, biosynthesis of various plant secondary metabolites, arginine and proline metabolism, alpha‐linolenic acid metabolism, linoleic acid metabolism, arachidonic acid metabolism, valine, leucine and isoleucine biosynthesis, purine metabolism, phenylalanine, tyrosine and tryptophan biosynthesis, glycerophospholipid metabolism and aminoacyl‐tRNA biosynthesis (Figure S9E). Furthermore, in CK vs PbNP, the top 20 significantly enriched pathways were linoleic acid metabolism, amino sugars, nucleotide, glycerophospholipid, arachidonic acid, vitamin B6, cysteine, purine, methionine, glyoxylate, dicarboxylate, glucuronate interconversions, lysine degradation, alpha‐linolenic metabolisms and biosynthesis of various plant secondary metabolites, pentose phosphate pathway, phenylalanine, tyrosine and tryptophan biosynthesis, aminoacyl‐trna biosynthesis and ABC transporters (Figure S9F). Last, in CK vs PbNPSM group, the top 20 significantly enriched pathways were pyrimidine, galactose, glycerophospholipid, linoleic acid, arachidonic acid, beta‐alanine, tryptophan, vitamin B6, arginine, proline, alpha‐linolenic acid metabolism, flavone, flavonol, flavonoid, glucosinolate, betalain biosynthesis, diterpenoid, secondary metabolites biosynthesis, lysine degradation, and ABC transporters (Figure S9G). Furthermore, integrated transcriptomic and metabolomic profiling highlighted that the key DEGs and DAMs were predominantly associated with several critical pathways, including ABC transporters, purine metabolism, flavonoid biosynthesis, and the synthesis of various secondary metabolites (Figure 6).

## 4 Discussion

In this study, the effects of single and combined treatments with Pb and NP on the growth, oxidative stress, transcriptome, and metabolite responses of rice seedlings were explored. The results showed that the individual or combined stress of Pb and NP significantly impaired rice growth, antioxidant responses, hormone synthesis, transcriptome, and metabolite profiles. In contrast, seed treatment with *M. anisopliae* caused significant changes in these parameters, ultimately improving plant growth. The findings also revealed a “Trojan Horse” effect, where negatively charged NP acted as carriers to facilitate Pb entry into plant tissues, causing severe growth inhibition observed in the PbNP treatment group [12, 32], whereas sowing of rice seeds soaked in negatively charged *M. anisopliae* conidia possibly disrupted this synergistic effect, resulting in reduced phytotoxicity to plants.

Our findings demonstrate a significant reduction in rice growth traits (plant height, root length, root fresh weight, and root dry weight) compared to the control under individual or combined stress of Pb and NP. These adverse effects on plant growth and development can be attributed in part to the root obstruction of nutrient uptake caused by high Pb application. Pb^2+^ ions compete with essential nutrients like Ca^2+^, Mg^2+^, and K^+^ for root absorption sites, leading to nutrient imbalances that compromise chlorophyll synthesis and overall plant vigor [33]. Furthermore, Pb phytotoxicity can cause microscopic tears in root cell walls and membranes, creating easier pathways for Pb ions to enter cytoplasm [34]. On the other hand, the phytotoxicity of Pb can be significantly increased under the co‐stress of Pb and NP, as Pb ions alone face specific barriers at root cell walls; being bound to nano‐sized plastics may allow them to bypass some regulatory mechanisms [35]. These findings align with previous studies reporting that the combination of plastics and heavy metals curtails plant growth and development [10, 12]. Jin et al. [12]. examined the effects of polypropylene plastics and lead on wheat seed germination, seedling growth, and antioxidant responses, and reported a similar role of plastics as carriers that enhance Pb mobility through surface adsorption and altered metal speciation. This also aligns with findings in *Fagopyrum esculentum* exposed to polylactic acid microplastics and Pb, where the plastic contamination increased Pb bioavailability by disrupting root membrane integrity and facilitating metal translocation to shoots, despite the microplastics being biodegradable [35]. It was observed that seed inoculation with *M. anisopliae* significantly enhanced the rice morphological growth and plant biomass production. These results were consistent with previous studies reporting that *M. anisopliae* colonize plants and enhance root growth and water and nutrient uptake, thereby improving growth [36]. These changes in growth parameters can be attributed to reduction in Pb as well as NP phtotoxicity through competitive adsorption and surface complexation interactions between fungal spores/mycelia (composed of chitin, chitosan, and glycoproteins rich in negatively charged functional groups like carboxyl, phosphate, amino, hydroxyl) having affinity for Free Pb^2+^ ions (via ion exchange and complexation) [37] as well as immobilization of negatively charged NPs electrostatic repusion and entanglement [38]. In this way, fungal treatment can create a powerful sink to strip Pb from the NP surface before it reaches the root, breaking the PbNP complex or adsorbing the entire PbNP complex onto its extensive mycelial network, physically preventing root contact [39].

Our results revealed that the toxicological impacts of Pb and NPs on rice, whether individually or in combination, lead to substantial chlorophyll degradation through distinct yet complementary mechanisms (as confirmed by transcriptomic analysis). This analysis showed that PbNP down‐regulated photosynthetic pathways and might also increase chlorophyll degradation, resulting in lower chlorophyll content. In isolation, Pb^2+^ ions directly inhibit key enzymes in the chlorophyll biosynthesis pathway, such as δ‐aminolevulinic acid dehydratase (ALAD) and protochlorophyllide reductase, and substitute magnesium (Mg^2+^) (the central ion in chlorophyll), resulting in the formation of non‐functional, Mg‐deficient porphyrins [40]. Independently, nanoplastics induce physical shading, obstruct stomatal apertures, and generate oxidative stress that degrades existing chlorophyll and damages thylakoid membranes, thereby reducing light‐harvesting efficiency [41]. However, when both stressors are present, these effects are markedly intensified: the “Trojan Horse” phenomenon, in which NPs facilitate increased Pb uptake, leads to excessive chlorophyll loss. This combined stress not only inhibits biosynthetic enzymes but also accelerates ROS‐mediated photo‐oxidation of chlorophyll molecules and severely disrupts chloroplast ultrastructure, including grana stacking and thylakoid membrane integrity [12]. However, *M. anisopliae* treatment enhanced the chlorophyll synthesis in the NPSM, PbNP, and PbNPSM groups. In this study, mitigating the individual or combined toxicity of Pb and NPs through rhizosphere *M. anisopliae* seed dressing is expected to enhance plant chlorophyll levels through a comprehensive recovery process significantly. This enhancement primarily stems from reduced direct lead toxicity, as decreased Pb2+ uptake alleviates its inhibition of essential enzymes in chlorophyll biosynthesis [42]. Concurrently, by mitigating the synergistic oxidative surge, the intervention reduces reactive oxygen species (ROS)‐induced degradation of the chlorophyll porphyrin ring and protects thylakoid membranes from lipid peroxidation [43]. Furthermore, the sequestration of Pb in the rhizosphere diminishes its competition with magnesium (Mg^2+^) and iron (Fe), thereby restoring the availability of these vital nutrients for chlorophyll synthesis and electron transport [44]. Last, safeguarding chloroplast ultrastructure from nanoplastic‐induced physical damage and reinstating nitrogen metabolism to supply precursors creates a conducive environment for chlorophyll assembly [45]. Collectively, these processes counteract the co‐stress damage by enhancing both the stability of existing chlorophyll and the rate of new biosynthesis, resulting in significant increases in chlorophyll a, b, and total content, which serve as comprehensive indicators of restored photosynthetic health.

In the present study, individual or combined stress of Pb and NP significantly affected the activities of antioxidant enzymes (SOD, POD, CAT) and the production of reactive oxygen species (ROS). The combined and individual phytotoxic effects of lead (Pb) and nanoplastics (NPs) synergistically disrupt plant redox balance, resulting in a significant oxidative burst that overwhelms antioxidant defenses. When present independently, Pb^2+^ ions induce the production of reactive oxygen species (ROS) by disrupting mitochondrial electron transport and deactivating antioxidant enzymes such as catalase (CAT) and ascorbate peroxidase (APX) through metal binding [46]. Nanoplastics, in isolation, cause physical damage to membranes and generate ROS through surface oxidative potential, often leading to an initial increase in superoxide dismutase (SOD) and peroxidase (POD) activities as a compensatory mechanism [47]. However, when both are present, their interaction results in a supra‐additive effect: NPs facilitate increased Pb uptake (“Trojan Horse” effect). At the same time, Pb adsorbed on NP surfaces catalyzes Fenton‐like reactions, producing highly destructive hydroxyl radicals (−OH) that antioxidant systems cannot efficiently neutralize [48]. This combined stress results in a paradoxical failure of enzyme efficacy (where SOD activity may initially rise and then fall due to metal‐induced cofactor displacement, and glutathione reserves become depleted), leading to irreversible oxidative damage to lipids, proteins, and DNA. However, the above‐mentioned changes in antioxidant enzyme activity (SOD, POD, and CAT) and the production of reactive oxygen species (ROS) were substantially mitigated by *M. anisopliae* seed inoculation in the NPSM, PbNP, and PbNPSM groups. The competitive adsorption and surface complexation interactions between *M. anisopliae* spores, Pb, and NPs are responsible for disrupting the individual or combined stress of Pb and NPs, rebalancing the plant's oxidative stress response, shifting from overwhelmed defense to managed homeostasis. With reduced uptake of Pb^2+^ and internalization of nanoplastics, the primary causes of synergistic ROS generation are significantly lessened. Consequently, the production of superoxide (O^2−^), hydrogen peroxide (H_2_O_2_), and hydroxyl radicals (−OH) decreases to baseline levels, alleviating the severe oxidative burst that causes lipid peroxidation and macromolecular damage [49]. This reduced ROS load allows the plant's antioxidant enzyme system to recover from suppression and hyperactivation; specifically, the activities of SOD, CAT, and POD transition from a stress‐induced, often inefficiently high state back to optimized, constitutive levels necessary for normal metabolic regulation [50]. Furthermore, the lowered cellular damage reduces the demand for non‐enzymatic antioxidants (e.g., glutathione, ascorbate), allowing these pools to regenerate. Mechanistically, this recovery is driven by the downregulation of ROS‐generating NADPH oxidases (RBOH genes) and the restoration of metal homeostasis (particularly Fe and Cu, cofactors for antioxidant enzymes), thereby preventing Fenton‐like reactions catalyzed by free Pb [46]. Ultimately, breaking the Trojan Horse effect restores the redox equilibrium, where antioxidant enzyme activity is sufficient to scavenge baseline metabolic ROS, thereby preventing oxidative signaling from escalating into oxidative stress and enabling normal growth and development.

Since the growth recovery post *M. anisopliae* seed treatment under individual or co‐stress of Pb and NP requires coordinated regulation of metabolism and signaling, we further examined the role of phytohormones (GA, IAA, JA, and SA), as plant responses to stress conditions are regulated by hormone synthesis. Phytohormones (GA, IAA, JA, and SA) are essential hormones that play an imperative role in root and shoot growth [51]. The decrease in root growth observed in the present study could be directly linked to reduced synthesis under individual or combined stress from Pb and NP. Furthermore, the high Pb concentration likely directly interfered with IAA transport and signaling, compounding the growth inhibition. Under conditions of individual or combined Pb and NP stress, an oxidative burst and nutrient disruption can initiate a typical stress hormone cascade: abscisic acid (ABA) levels increase, resulting in stomatal closure and the expression of stress‐responsive genes, while ethylene (ET) and jasmonic acid (JA) accumulate, promoting senescence and defense mechanisms at the expense of growth [52]. Concurrently, lead (Pb) interference with tryptophan metabolism and PIN transporter function suppresses the biosynthesis and polar transport of indole‐3‐acetic acid (IAA), and gibberellin (GA) levels decrease due to oxidative inactivation [53]. Our results showed that *M. anisopliae* seed inoculation substantially increased the production of phytohormones (GA, IAA, JA, and SA) in NPSM, PbNP, and PbNPSM groups. Mechanistically counteracting the individual or combined Pb and NP stress reverses this imbalance: reducing oxidative stress downregulates the key ABA biosynthetic enzyme NCED. It decreases ACC synthase activity, thereby reducing ET production [54]. With metal homeostasis restored, the enzymatic pathways for IAA (via YUCCA) and GA (via GA20‐oxidase) are reactivated, and enhanced photosynthetic capacity boosts the supply of carbon skeletons for hormone synthesis [55]. Earlier, various authors reported that endophytic fungi increase the synthesis of hormones (GA and IAA), thereby promoting plant growth by reducing stress ethylene through ACC deaminase activity [51]. This hormonal shift from a stressed to a balanced state encourages root elongation, cell division, and shoot development, ultimately restoring plant growth and productivity.

Soil microbes are a crucial component of the soil ecosystem [56]. In the present study, individual or combined stress of Pb and NP significantly altered the composition of the soil microbiota. Our results revealed a reduction in microbial diversity of Actinobacteria, Gammaproteobacteria, and Bacilli under the Pb treatment group compared to the control. The changes can be explained by the fact that when acting independently, Pb exerts an intense selective pressure, reducing microbial diversity and biomass while favoring metal‐resistant but often less beneficial plant taxa, such as certain Actinobacteria, and inhibiting crucial mutualists like nitrogen‐fixing bacteria (Cyanobacteria and phosphate‐solubilizing microbes (Gammaproteobacteria, Betaproteobacteria, Actinobacteria, Bacilli) [57]. In NP treatment group, microbial diversity of Alphaproteobacteria, Gammaproteobacteria, Actinobacteria, Bacilli was higher than control. These results are consistent with the findings of Fei et al. [58]. who reported that NP stress alone can induce physical disturbances and alter soil microenvironments, leading to decreased bacterial richness and the proliferation of plastic‐degrading or hydrophobic surface‐colonizing microorganisms, often at the expense of generalist decomposers. Our results revealed a reduction in microbial diversity of Actinobacteria, Gammaproteobacteria, and Bacilli in the PbPN treatment group compared to the control. These results are consistent with Wang et al. [48]. who reported intensified disruption of microbial communities under metal‐NP co‐stress, resulting in a simplified microbial community with decreased functional redundancy, characterized by a plant growth‐promoting rhizobacteria (PGPR) collectively undermining essential soil functions such as nutrient cycling and disease suppression. Seed treatment with *M. anisopliae* effectively reversed the aforementioned trend and enriched the key beneficial bacterial genera in the PBSM, NPSM, and PbNPSM treatment groups. The application of *M. aniopliae* mitigates this phytotoxicity by initiating a targeted restoration of the microbiome through various mechanisms. The introduced fungus acts as a “foundation species”, directly inhibiting pathogenic microbes through competition and antibiosis. At the same time, its exudates (e.g., organic acids, polysaccharides) form a recalcitrant carbon sink that selectively encourages the recovery of beneficial, copiotrophic bacteria [59]. Additionally, by immobilizing Pb and sequestering NPs, *M. aniopliae* can alleviate selective metal/particle stress on the native microbial community, thereby facilitating the natural reassembly of a more diverse and functionally resilient community [60]. This fungal‐driven restoration of a plant‐beneficial microbiome (characterized by an increased abundance of PGPR and N‐cycling bacteria) enhances nutrient bioavailability. It induces systemic resistance in the plant, thereby breaking the stress cycle and creating a positive feedback loop for ecosystem recovery.

Here, the different metabolic pathways in KEGG were also analyzed for their effects under other treatments. It was observed that functions related to signal transduction, lipid transport, transcription, and amino acid synthesis and transport were enhanced under PbNP and were further enriched by *M. anisopliae* inoculation. This suggested that the dual effects of Pb and NP altered metabolic pathways and soil microbial activity. Earlier, Ren et al. [61]. also found that MPs changed the abundance of amino acid metabolism, signal transduction, and lipid transport, thereby affecting plant physiological and biochemical functioning. *M. anisopliae* inoculation significantly enhanced the abundance of *Burkholderia‐Caballeronia‐Paraburkholderia*, *Mucilaginibacter*, *Sphingomonas*, and *Nocardioides*. The increase in the abundance of these bacteria could be linked with an increase in metabolic functioning and a reduction in Pb and NP owing to their fixation in soil after *M. anisopliae* inoculation. The increased relative abundance of growth‐promoting bacteria with *M. anisopliae* treatment can enhance plant growth under stress [62]. It is well reported that fungal inoculation maintains and balances the distribution of nutrients in the micro‐environment through its mycelial network, thereby increasing nutrient absorption by the host and, consequently, the resource availability for microbial communities [63]. Further, fungal inoculation also enhances the adaptability and prowess of soil bacteria [64]. Therefore, the increase in the abundance of favorable bacteria following *M. anisopliae* treatment was linked to its potential to enhance the adaptability and prowess of the soil bacterial community. It is important to note that this study focused on the bacterial (16S) community profile. Future studies incorporating ITS sequencing of the soil fungal community are essential to provide a holistic view of the microbiome. This would allow direct tracking of the inoculated *M. anisopliae* and reveal how the native fungal community, including potential plant symbionts and pathogens, responds to the combined stress and treatment, offering deeper mechanistic insights.

Gene expression is an extensive set of plant responses to stress conditions. Regarding the regulation of more functionally DEGs, co‐exposure to PbNP results in greater alterations than their individual exposure to each factor. Previous studies also reported more alterations in DEGs under combined MPs and cadmium (Cd) toxicity than their individual toxicity [65]. The individual and combined stresses of lead (Pb) and nanoplastics (NPs) induce substantial upregulation of genes encoding oxidative stress‐responsive proteins, heavy‐metal chelators, and pathogenesis‐related proteins. In contrast, genes essential for photosynthesis, cell division, and primary metabolism are downregulated [66]. The reduced carbon fixation was responsible for the reduction in rice growth under combined and alone Pb and NP stress. Furthermore, co‐exposure to Pb and NP also decreased carbohydrate metabolic pathways. This results in reduced production of glucose and maltose, which maintain cellular turgor pressure, act as osmo‐protectants, and scavenge ROS [67]. The results indicated that DEGs associated with signaling perception, transduction, phyto‐hormone synthesis, antioxidant defense, and secondary metabolite synthesis were up‐regulated in response to PbNP stress. This suggests that plants activated these pathways to counteract PbNP toxicity. Additionally, seed inoculation with *M. anisopliae* alters the DEGs involved in antioxidant defense mechanisms, biosynthesis of various plant secondary metabolites, ABC transporters, hormonal signaling, purine metabolism, and flavonoid biosynthesis, thereby conferring resistance to the individual or combined toxicity of Pb and NPs [68]. Crucially, this modulation of gene expression underpins the observed reduction in Pb uptake and the enhancement of detoxification pathways. Based on our findings, significant DEGs were upregulated under PbSM, NPSM, and PbNPSM, indicating that *M. anisopliae* seed inoculation mitigates the toxicity of Pb and NPs by upregulating several genes. The fungus acts as a biological signal modulator, as evidenced by the reduction in hyper‐induced oxidative stress and defense transcriptome, alongside the reactivation of genetic programs related to growth and development [45]_._ This is achieved through several intersecting mechanisms: fungal sequestration of stressors diminishes the activation signal for stress‐responsive transcription factors (such as WRKYs, MYBs); fungal‐derived molecular patterns (such as chitin) prime the plant's immune system, resulting in a more efficient and less resource‐intensive defense response; and fungal production of phytohormones (such as auxin) or modulation of plant hormone synthesis directly affects the expression of key developmental genes [59, 69]. Consequently, the transcriptomic profile transitions from a state of emergency defense to one of managed homeostasis and recovery, characterized by the restored expression of photosynthetic machinery, nutrient transporters, and cell wall biosynthesis genes, which support the observed physiological recovery in growth and chlorophyll content.

Metabolomics analysis revealed significant alterations in sugar metabolism and organic synthesis under individual or combined stress from Pb and NPs. The results showed that MPs and Pb stress disrupted rice metabolic pathways, leading to significant alterations in metabolite synthesis. Amino acids play a crucial role in physiological processes [70], and their synthesis was significantly altered by the individual and combined toxicity of Pb and NP. This occurs as central metabolic resources, including carbohydrates such as sucrose and glucose and essential amino acids such as glutamate and aspartate, are depleted due to impaired photosynthesis and disrupted nitrogen assimilation [71]. Conversely, NP stress alone leads to the accumulation of oxidative stress markers, such as MDA and oxidized glutathione, as well as secondary metabolites, such as flavonoids and phenolic acids, that function as antioxidants. It also disrupts lipid metabolism and causes osmolytes to leak from the cell [72]. When both stressors are present, their effects combine synergistically, resulting in a metabolic crisis characterized by a severe depletion of energy‐rich compounds such as ATP and NADPH, a significant bottleneck in TCA cycle intermediates, and an excessive accumulation of cytotoxic aldehydes and ROS‐damaged metabolites, exceeding the sum of their individual impacts [73, 74]. The KEGG pathways also showed the disruption in metabolic pathways needed for amino acid biosynthesis, serine, glycine, and arginine biosynthesis. In this study, *M. anisopliae* inoculation up‐regulated pathways involved in phenylpropanoid, lignin, flavonoid, and secondary metabolite biosynthesis. Amino acid synthesis was also increased after *M. anisopliae* treatment in the PbSM, NPSM, and PBNPSM treatment groups, indicating a comprehensive metabolic recovery by the fungus. *M. anisopliae* can serve as an external metabolic buffer by exuding chelating siderophores and organic acids that immobilize Pb and NPs, thereby reducing the plant's need for internal metal chelation and antioxidant production [75]. This allows the plant to redirect resources from costly stress responses back to primary metabolism. Fungal symbiosis enhances nitrogen and phosphorus uptake, replenishes amino acid and nucleotide pools, and can directly supply growth‐promoting metabolites, such as indole‐3‐acetic acid (IAA), to the plant [7]. Consequently, the plant's metabolic profile transitions from emergency catabolism and stress compound accumulation to anabolic recovery, characterized by the replenishment of carbohydrates, normalization of the TCA cycle, and a balanced, sustainable production of protective secondary metabolites, collectively restoring cellular homeostasis and growth [19].

## 5 Conclusion

**FIGURE 7:**
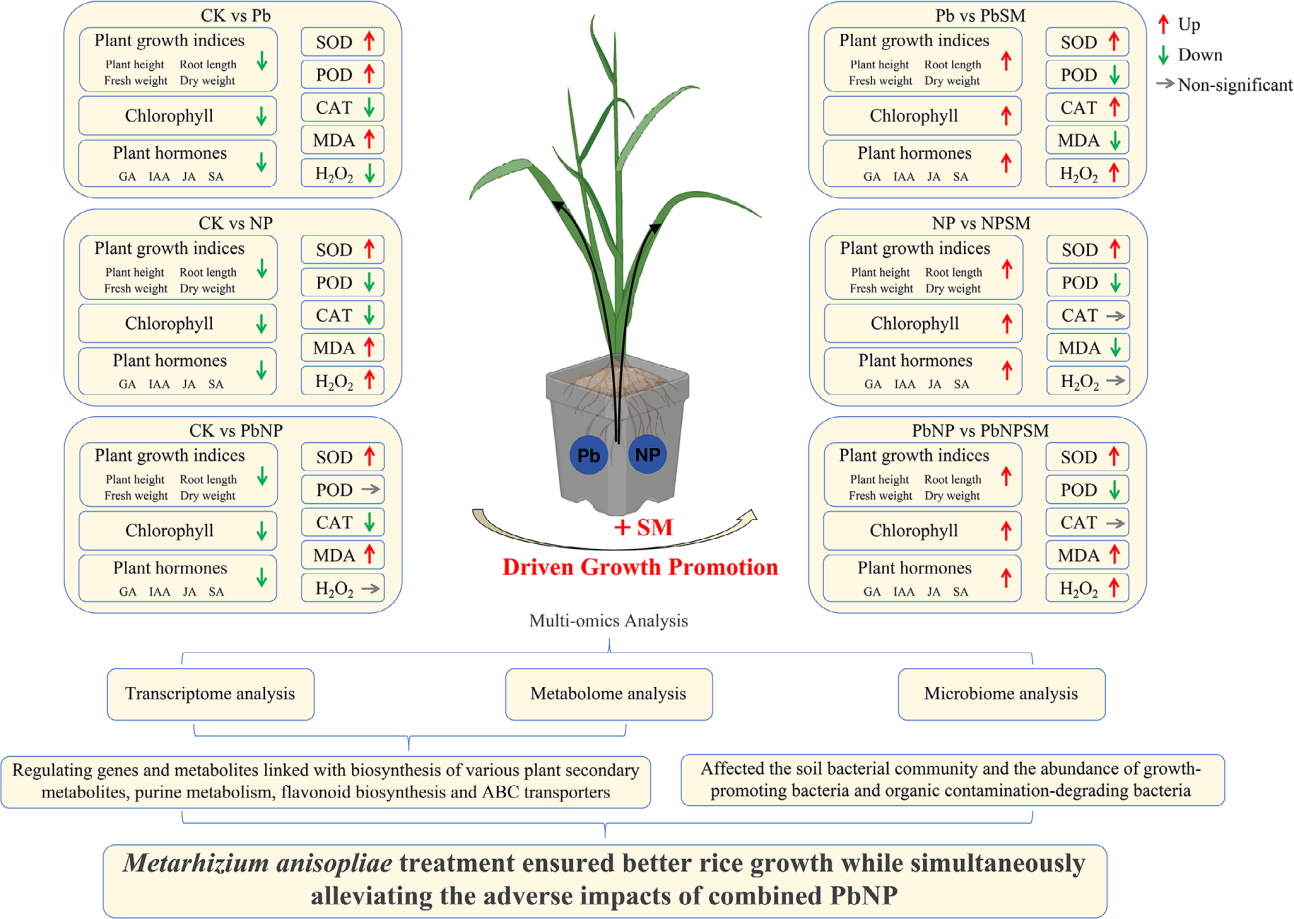
Model of the regulatory mechanism of *M. anisopliae* SM021 in mitigating the combined toxicity of lead and microplastics in rice. Red arrows indicate upregulation, green arrows indicate downregulation, and grey arrows indicate no significant change.

This study demonstrates that the individual or combined stress of Pb and NP significantly reduced rice growth by enhancing oxidative stress, disrupting antioxidant activity, hormone and metabolite synthesis, and the abundance of metal‐degrading bacteria. However, the strategic use of *M. anisopliae* seed treatment improved rice growth while simultaneously mitigating the adverse effects of combined PbNP (Figure 7). Seed treatment with *M. anisopliae* improved the plant physiological and biochemical responses, thereby leading to better growth in PbNP‐contaminated soil. The improved growth was also achieved by improved transcriptomic and metabolic processes related to antioxidant defense mechanisms, plant secondary metabolites, purine metabolism, photosynthesis, flavonoid biosynthesis, plant hormone signal transduction, and ABC transporters. *M. anisopliae* seed treatments also reshaped the soil bacterial community and abundance of growth and metals‐degrading bacteria, thereby improving soil quality and rice growth. This research provides the basis for a comprehensive understanding of the effects of individual or combined stress of Pb and NP on rice and their mitigation through *M. anisopliae* seed treatment. Thus, *M. anisopliae* shows potential to mitigate PbNP toxicity and enhance rice productivity in multi‐contaminated soils. However, further identification and characterization of key genes, metabolites, or proteins involved in this process are required to expand the scope of such research.

## Author Contributions

J.P. was responsible for conceptualization, methodology, data curation, and writing the original draft. Q.Y. contributed to conceptualization, investigation, methodology, and software development. M.U.H. performed visualization and data curation and prepared the original draft. M.I. carried out data curation, formal analysis, and software development. F.U.H. contributed to visualization and writing through review and editing. J.L. was involved in investigation and methodology. X.W. contributed to funding acquisition and resources. S.A. led conceptualization and methodology, provided resources, and contributed to writing through review and editing.

## Funding

Modern Agricultural Technology Industry System of Guangdong province 2024CXTD11. [Correction added on 2 March 2026 after online publication: Shandong is replaced with Guangdong in Funding section.]

## Conflicts of Interest

The authors declare no conflicts of interest.

## Supporting information




**Supporting File**: advs74243‐sup‐0001‐SuppMat.pdf.

## Data Availability

The data that support the findings of this study are available from the corresponding author upon reasonable request.
