# Supplementary material for: Metarhizium anisopliae Mitigates the Phytotoxicity of Lead and Nanoplastics on Rice by Modifying Physiological, Transcriptomic, Metabolomic Activities, and Soil Microbiome
Source: Adv Sci (Weinh). 2026 Feb 6;13(21):e21570. doi: 10.1002/advs.202521570 (PMC13073243; doi:10.1002/advs.202521570)
Supplement: Supplementary file 1 — Supporting File: advs74243‐sup‐0001‐SuppMat.pdf. [file ADVS-13-e21570-s001.docx]

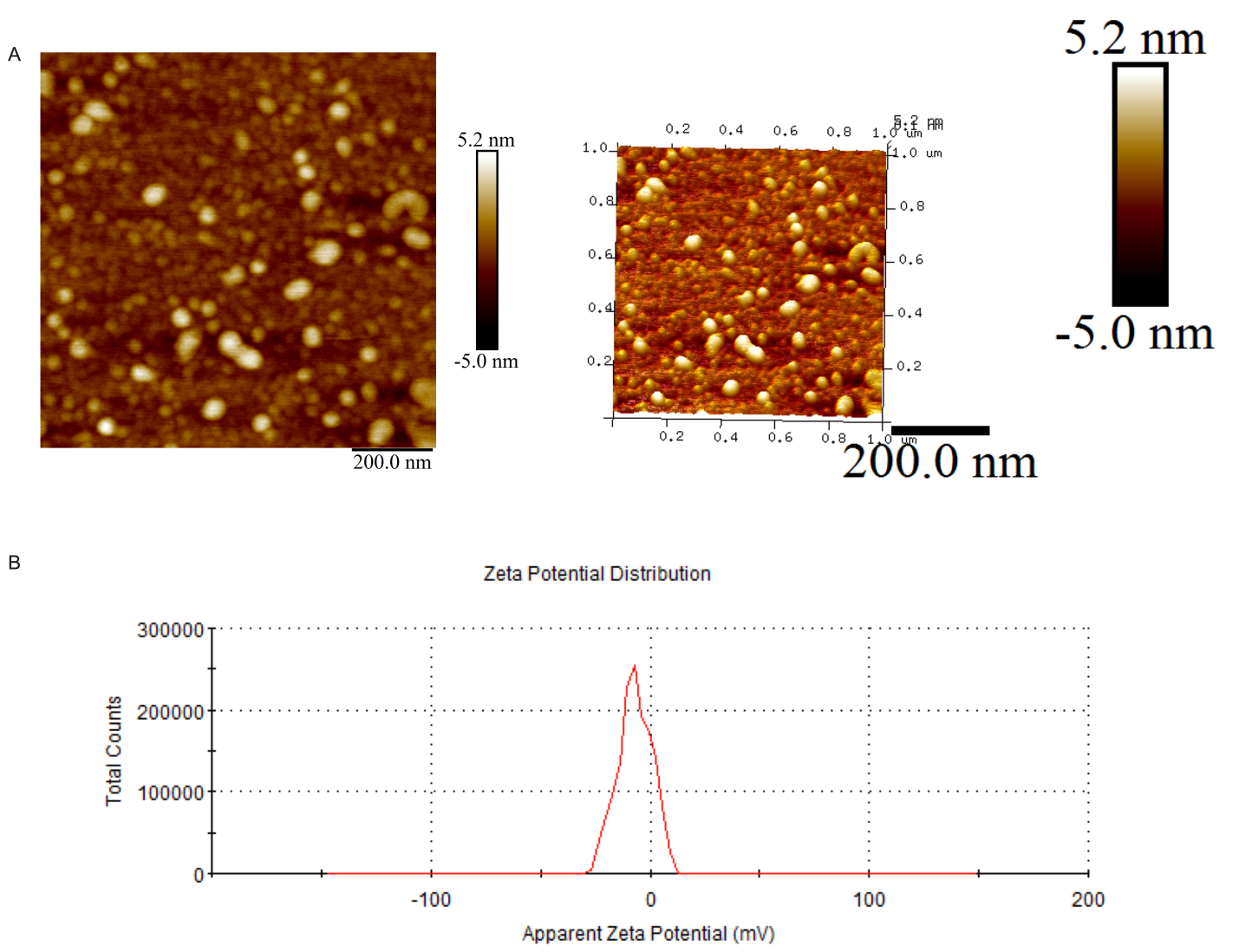


Figure S1. (A) Two-dimensional atomic force microscopy and three-dimensional atomic force microscopy of NP-MPs. (B) Particle size analysis of NP-MPs.


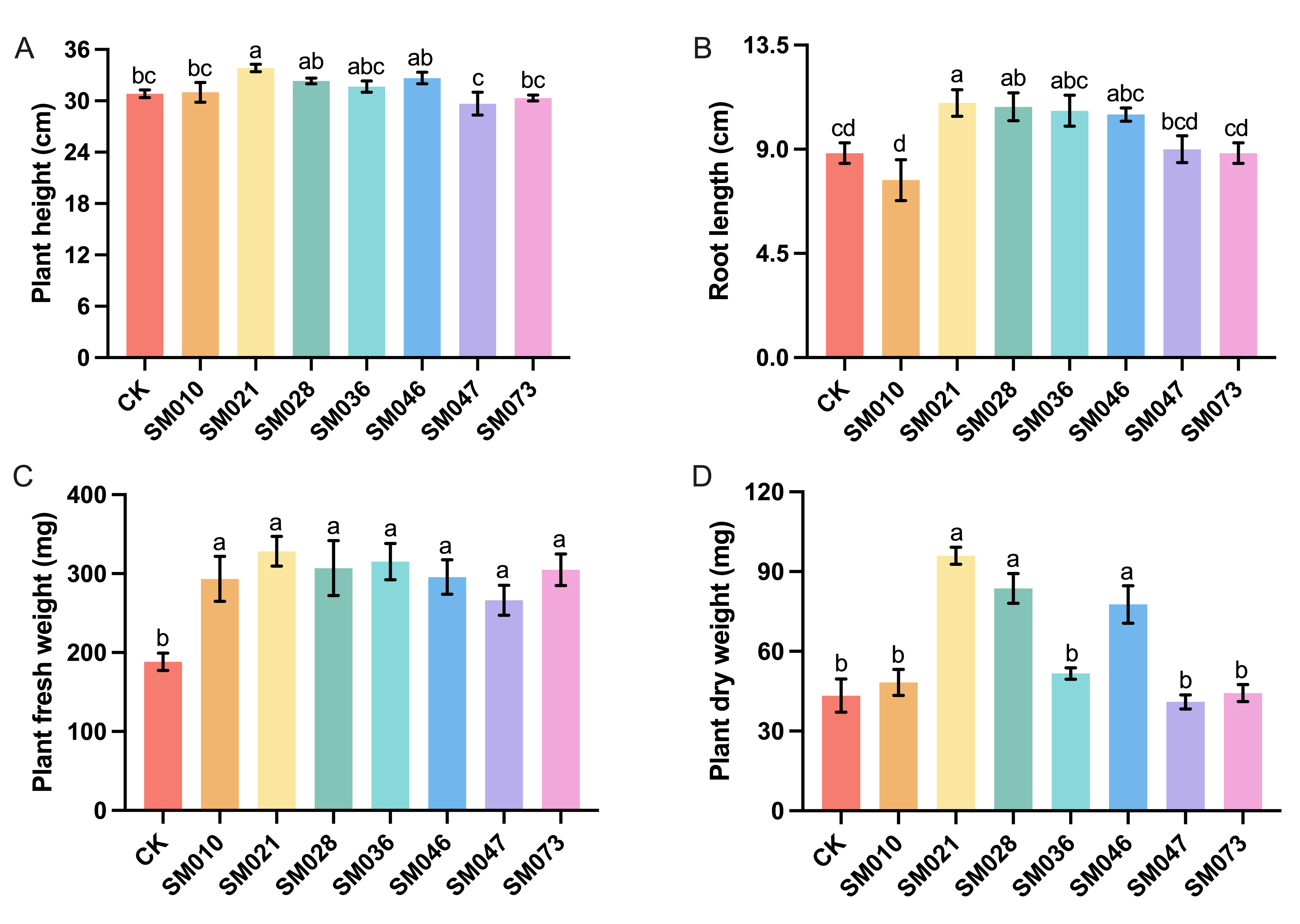


Figure S2. Effects of seven *M. anisopliae* strains on rice plant height (A), root length (B), plant fresh weight (C) and plant dry weight (D).

Data represent mean ± SE from three replicates per treatment. n=3. Different lowercase letters indicate statistically significant differences among treatments at *P* < 0.05.


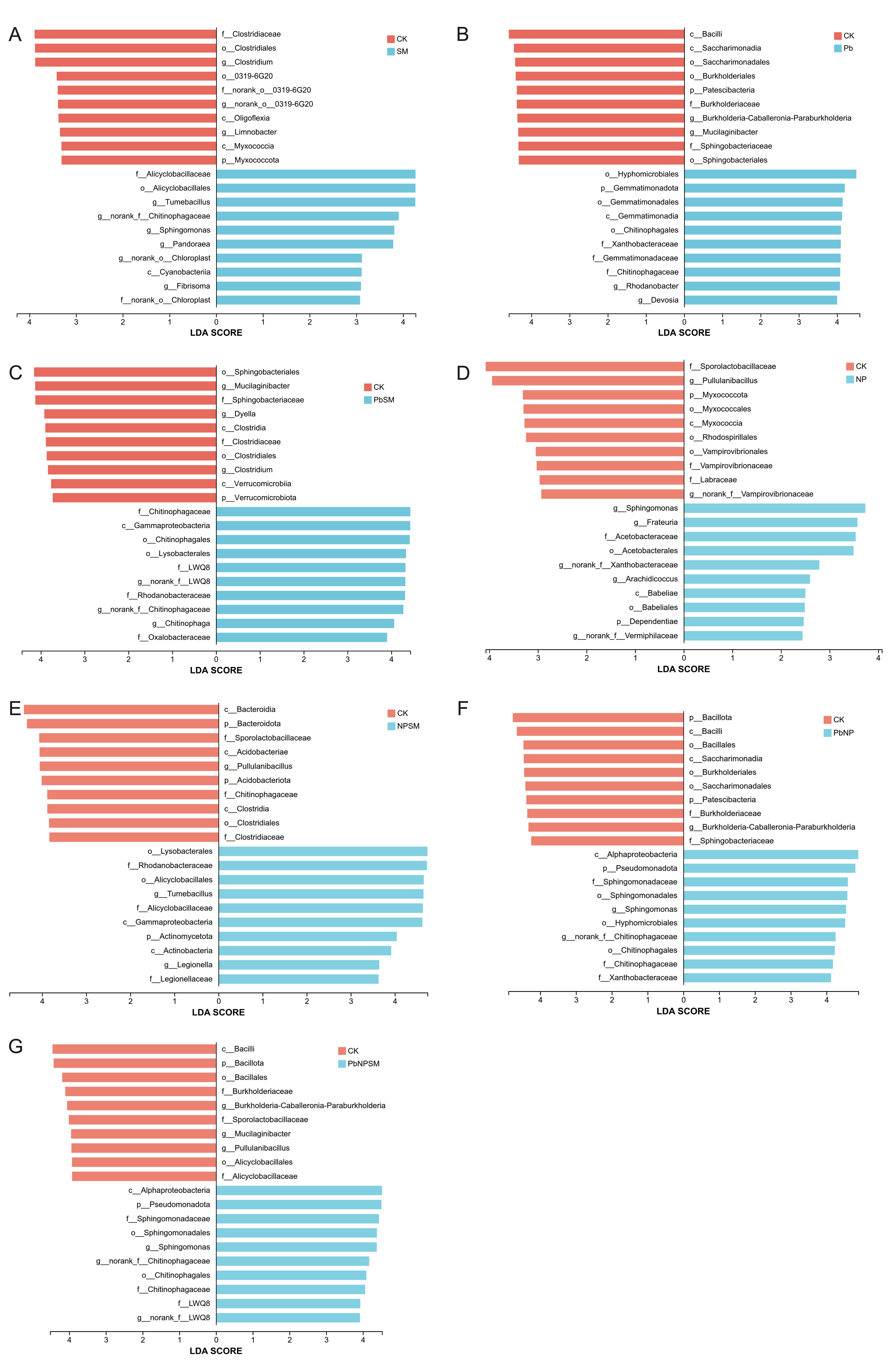


Figure S3. Microbial biomarkers identified by linear discriminant analysis (LDA) effect size (LEfSe).


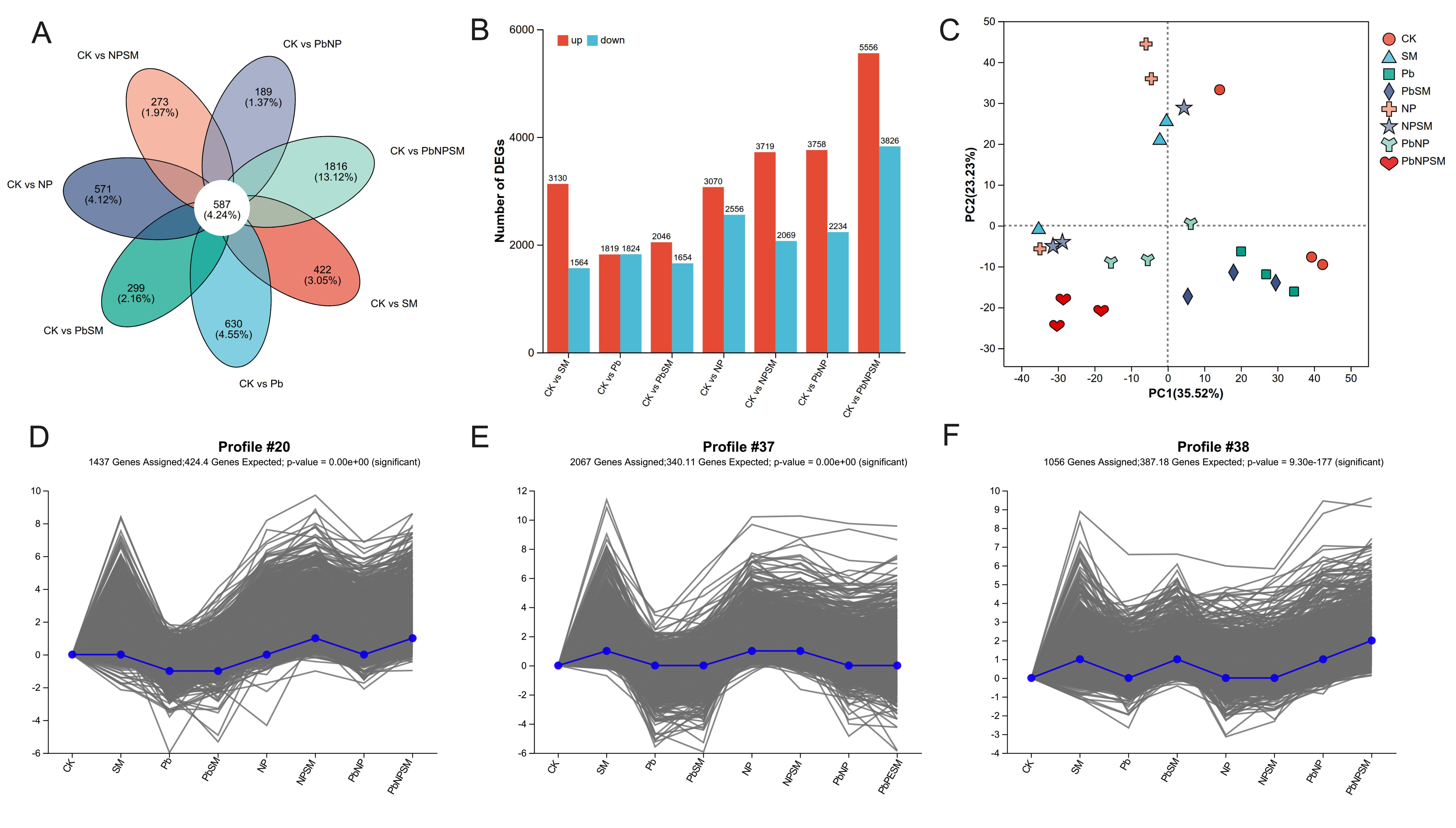


Figure S4. Trend analysis of DEGs under different treatments. The date is showing expression changes.


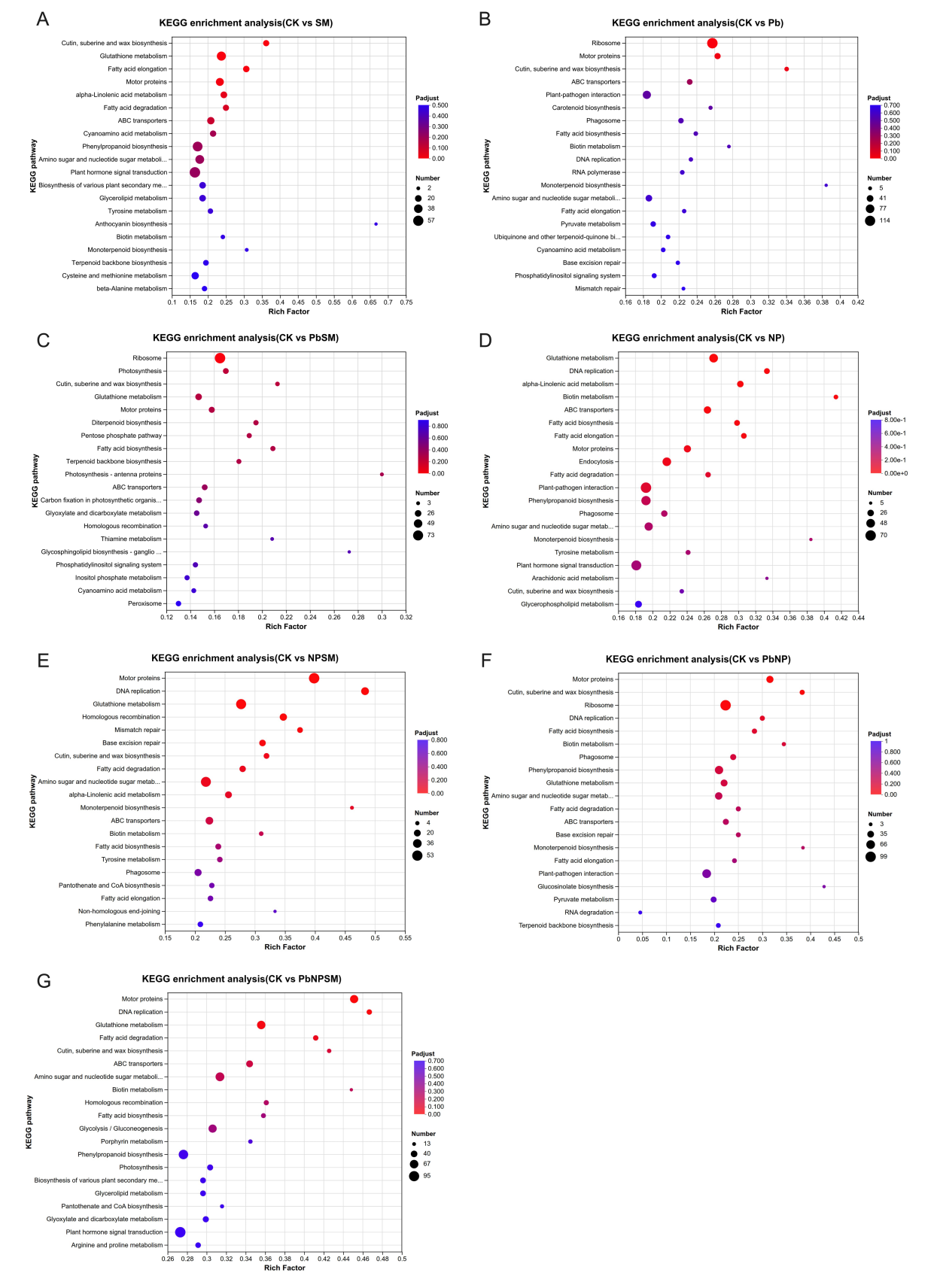


Figure S5. KEGG enrichment analysis of DEGs under different treatments. The y-axis shows pathway names, the x-axis shows Rich factor (ratio of enriched genes to annotated genes; higher values indicate greater enrichment). Dot size represents gene counts in each pathway, while color corresponds to different p-adjusted ranges.


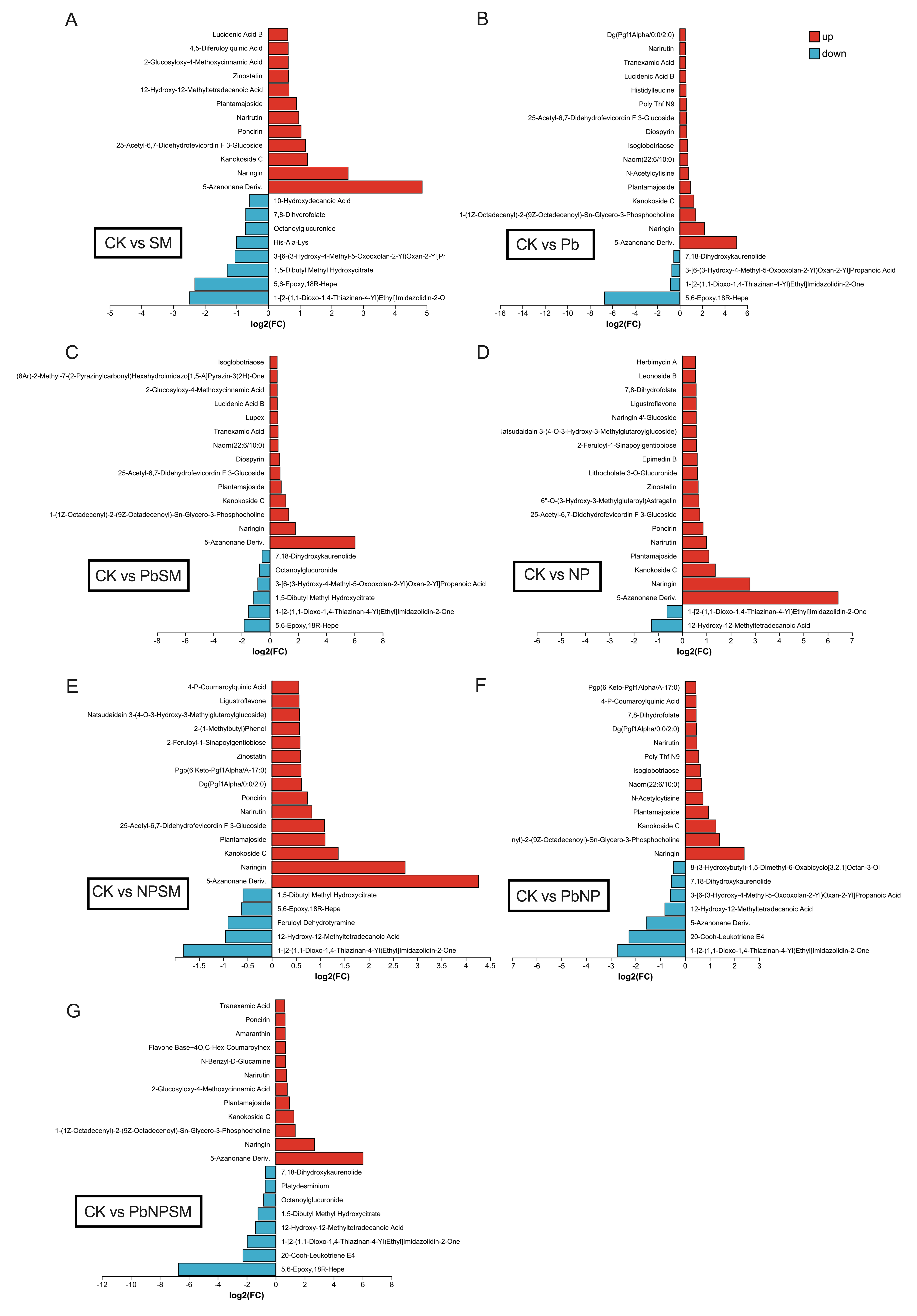


Figure S6. Expression analysis of top 20 DAMs by fold change. The x-axis shows log2FC, while the y-axis displays metabolite names.


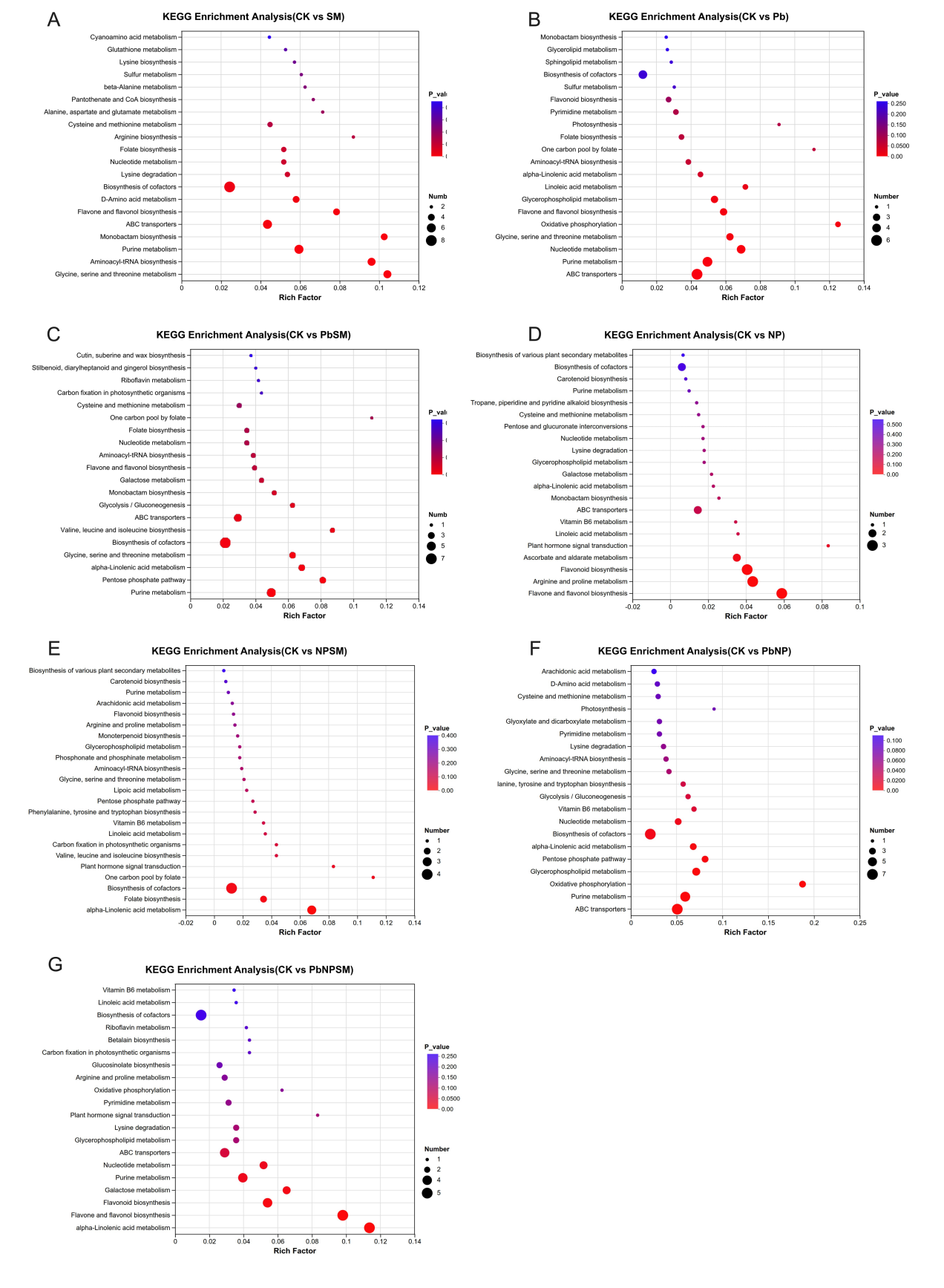


Figure S7. KEGG enrichment analysis of DAMs under different treatments. The y-axis shows pathway names, the x-axis shows Rich factor, dot size represents metabolite counts, and color corresponds to different p-adjusted ranges.


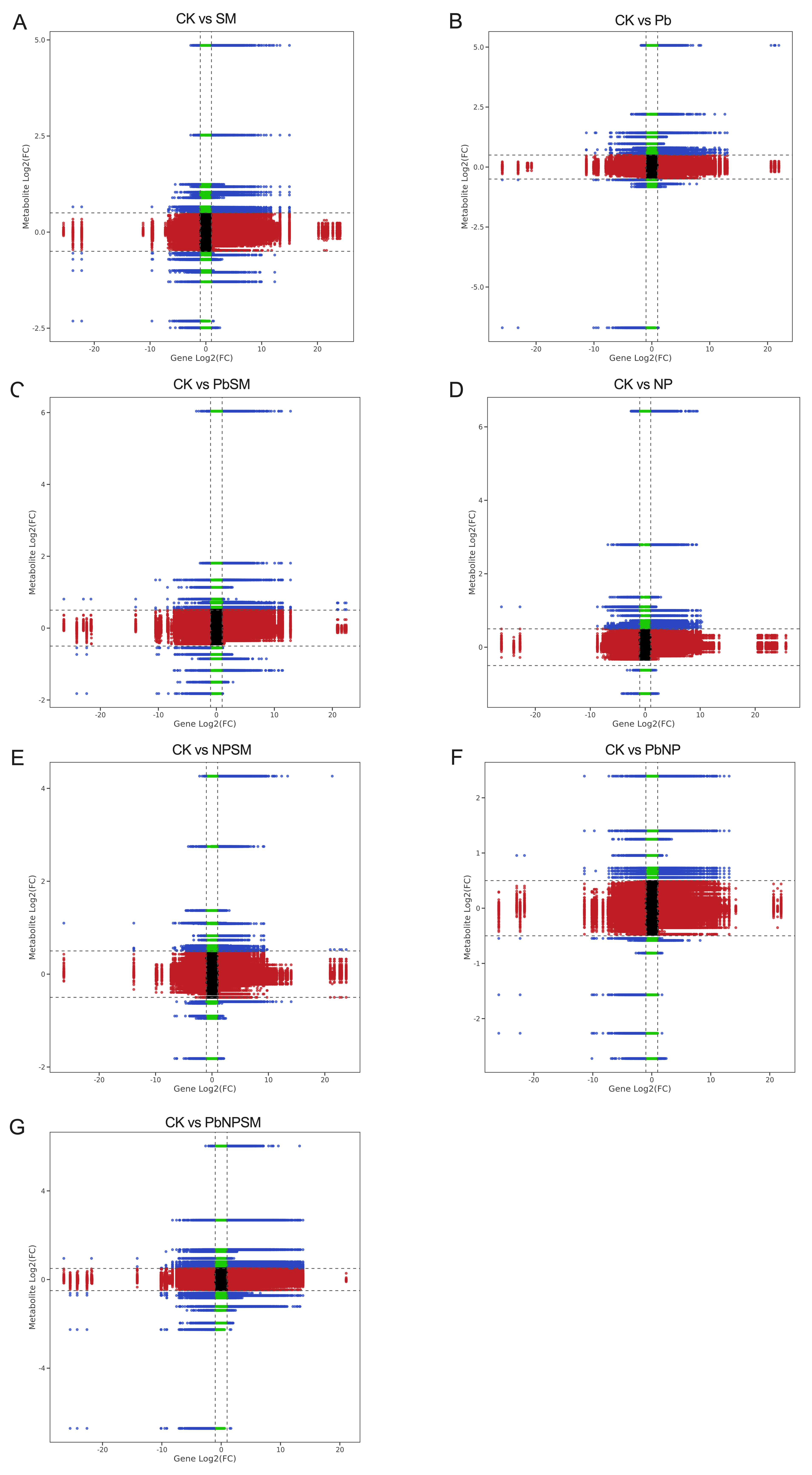


Figure S8. Nine-quadrant plots of DAMs and DEGs with Pearson correlation coefficients > 0.8. The plots display fold changes of metabolites and genes in each differential group.


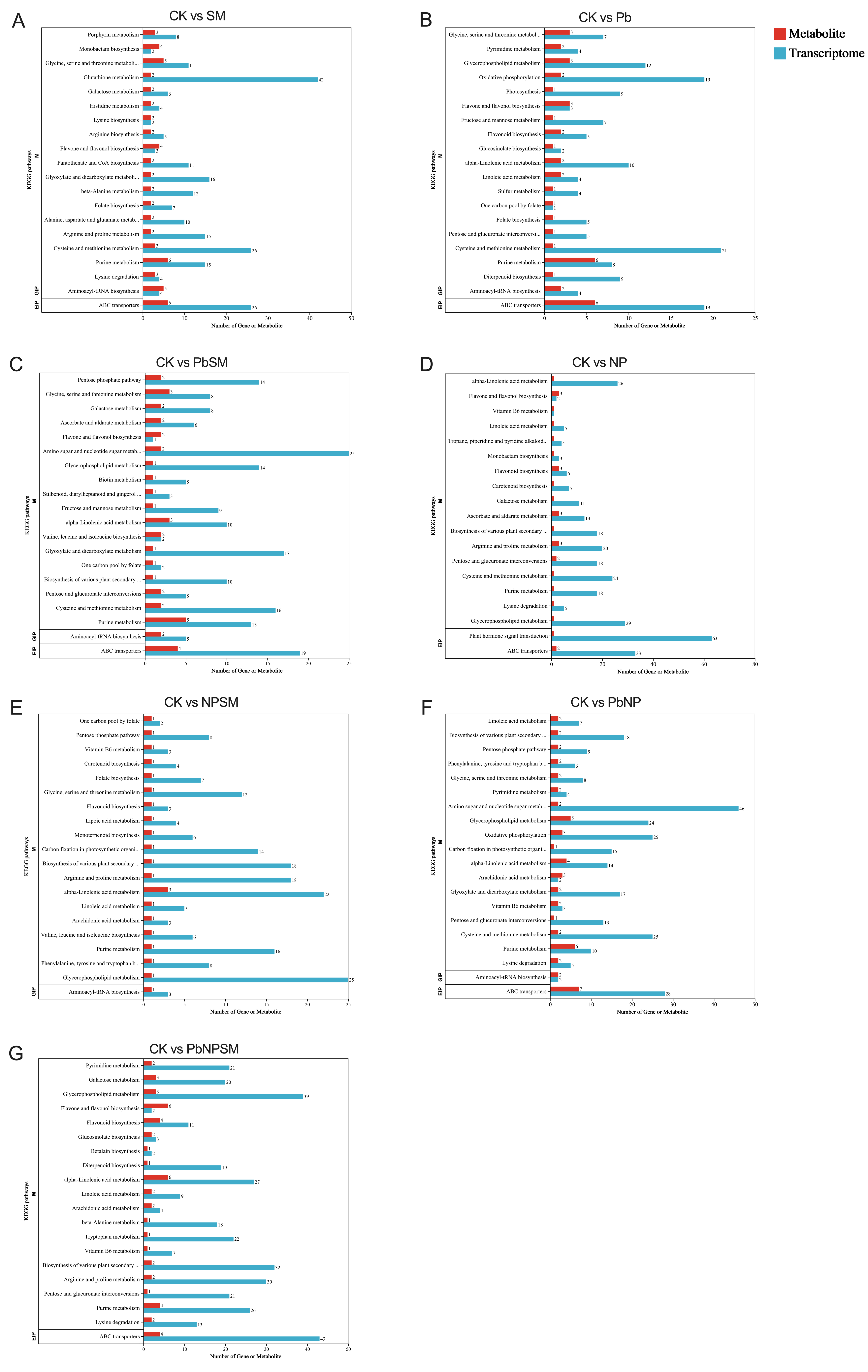


Figure S9. KEGG enrichment analysis of DEGs and DAMs co-enriched in the same pathways.

Table S1 Physical and chemical properties of soil

| Treatments | PH | Org matter (%) | Total N (%) | Plant available Pb (mg/kg) |
| --- | --- | --- | --- | --- |
| Pb | 5.27 ± 0.09 a | 46.75 ± 1.01 a | 3.53 ± 0.06 a | 22.72 ± 0.42 b |
| PbNP | 5.25 ± 0.09 a | 45.89 ± 1.30 a | 3.51 ± 0.03 a | 26.38 ± 0.39 a |
| CK | 5.32 ± 0.08 a | 46.23 ± 1.13 a | 3.48 ± 0.05 a | 1.02 ± 0.04 c |
| NP | 5.29 ± 0.13 a | 46.21 ± 1.3 a | 3.55 ± 0.04 a | 1.35 ± 0.03 c |

Table S2 Relative abundance of major microbial genera

|  | CK | SM | Pb | PbSM | NP | NPSM | PbNP | PbNPSM |
| --- | --- | --- | --- | --- | --- | --- | --- | --- |
| Burkholderia-Caballeronia-Paraburkholderia | 0.057338302 | 0.050057795 | 0.007194245 | 0.070743405 | 0.065593568 | 0.065507306 | 0.015035454 | 0.036557804 |
| Ammoniphilus | 0.042596139 | 0.077238928 | 0.024032573 | 0.024092956 | 0.047806359 | 0.054431275 | 0.03790349 | 0.047780481 |
| norank_f__Micropepsaceae | 0.039378569 | 0.03171851 | 0.040801891 | 0.038921381 | 0.040042786 | 0.028914997 | 0.052671532 | 0.058002519 |
| Mucilaginibacter | 0.060607629 | 0.049842141 | 0.016717561 | 0.031744389 | 0.05204182 | 0.036609561 | 0.025835447 | 0.040387834 |
| Sphingomonas | 0.017321395 | 0.030226179 | 0.032063558 | 0.014405742 | 0.030243431 | 0.009057502 | 0.089600262 | 0.065938616 |
| norank_f__Chitinophagaceae | 0.014121077 | 0.03135621 | 0.032003174 | 0.050187188 | 0.022246951 | 0.011817884 | 0.052033194 | 0.044071218 |
| unclassified_f__Sphingomonadaceae | 0.025128099 | 0.021763884 | 0.030622984 | 0.039663233 | 0.026758449 | 0.02904439 | 0.037894864 | 0.037032245 |
| Neobacillus | 0.033478253 | 0.039145662 | 0.036695823 | 0.022824906 | 0.042483998 | 0.020737367 | 0.021056536 | 0.028354295 |
| Tumebacillus | 0.015242482 | 0.060616255 | 0.000448562 | 0.008289771 | 0.031684005 | 0.107292583 | 0.002268689 | 0.000414057 |
| unclassified_o__Saccharimonadales | 0.042768663 | 0.042259717 | 0.001250798 | 0.031891034 | 0.021177303 | 0.051437986 | 0.003804151 | 0.022583372 |
| norank_f__LWQ8 | 0.014578265 | 0.013060056 | 0.021004779 | 0.05598399 | 0.013474113 | 0.014526508 | 0.020418198 | 0.033133206 |
| unclassified_f__Rhodanobacteraceae | 0.010472198 | 0.007616928 | 0.018908614 | 0.048789745 | 0.034634163 | 0.024308611 | 0.012896158 | 0.024731294 |
| Dyella | 0.025222987 | 0.021461967 | 0.016036092 | 0.009264531 | 0.018891362 | 0.060676639 | 0.014811173 | 0.014034815 |
| Pullulanibacilus | 0.038369305 | 0.024153339 | 0.002725877 | 0.049807636 | 0.016717561 | 0.015604782 | 0.005676035 | 0.022479858 |
| Devosia | 0.014492004 | 0.013758777 | 0.034358125 | 0.015509894 | 0.013715646 | 0.010799993 | 0.030191674 | 0.018115004 |
| unclassified_f__Comamonadaceae | 0.015121716 | 0.011421079 | 0.025024585 | 0.01037731 | 0.018451426 | 0.010869003 | 0.015863568 | 0.027112123 |
| Frateuria | 0.005115332 | 0.01461277 | 0.003968049 | 0.013646637 | 0.012507979 | 0.061271846 | 0.008332902 | 0.014215965 |
| Nocardioides | 0.012784017 | 0.016243121 | 0.003079551 | 0.022764522 | 0.015121716 | 0.030476338 | 0.01243897 | 0.009678588 |
| Edaphobacter | 0.013448234 | 0.013681141 | 0.00575367 | 0.017856219 | 0.01345686 | 0.010239291 | 0.015708297 | 0.018649828 |
| Ramlibacter | 0.012663251 | 0.010075393 | 0.016665804 | 0.014103825 | 0.019736729 | 0.008194883 | 0.015061332 | 0.007513414 |
| unclassified_f__Xanthobacteraceae | 0.010429067 | 0.006935459 | 0.02480893 | 0.010834498 | 0.007875714 | 0.006840571 | 0.021056536 | 0.012180184 |
| unclassified_f__Oxalobacteraceae | 0.012137053 | 0.010498076 | 0.009178269 | 0.0191674 | 0.008091369 | 0.018977624 | 0.012663251 | 0.009911495 |
| unclassified_f__Rhizobiaceae | 0.009126512 | 0.008358781 | 0.017726826 | 0.013784656 | 0.010351431 | 0.005632904 | 0.020366441 | 0.012326829 |
| norank_c__Sericytochromatia | 0.008022359 | 0.017856219 | 0.005598399 | 0.008712454 | 0.008953988 | 0.01703673 | 0.004476994 | 0.022410848 |
| Paenibacillus | 0.01189552 | 0.012861653 | 0.007987854 | 0.015164847 | 0.00987699 | 0.014733537 | 0.007616928 | 0.011636734 |
| unclassified_f__Bacillaceae | 0.014267723 | 0.009626831 | 0.015466763 | 0.010204786 | 0.021263565 | 0.005356866 | 0.007547918 | 0.004753032 |
| Massilia | 0.008669324 | 0.006624916 | 0.003079551 | 0.017838966 | 0.016864206 | 0.004321722 | 0.011274434 | 0.01533737 |
| Pseudolabrys | 0.008307023 | 0.006892328 | 0.016398392 | 0.008263892 | 0.005624278 | 0.007038973 | 0.017692321 | 0.009178269 |
| Chitinophaga | 0.006987216 | 0.00169936 | 0.005503511 | 0.030165795 | 0.01431948 | 0.002363577 | 0.003355589 | 0.007996481 |
| Rhodanobacter | 0.000103514 | 0.000319169 | 0.02596484 | 0.010291048 | 0.001526836 | 0 | 0.019253662 | 0.013629384 |
| others | 0.399806773 | 0.338017356 | 0.498930352 | 0.264703345 | 0.338465918 | 0.25587875 | 0.385194003 | 0.309852837 |
